# Loss of myeloid‐specific lamin A/C drives lung metastasis through Gfi‐1 and C/EBPε‐mediated granulocytic differentiation

**DOI:** 10.1002/mc.23147

**Published:** 2020-01-07

**Authors:** Hiroki Ishii, Woo‐Yong Park, Jaeyoung So, Skyler Kuhn, Vishal N. Koparde, Yanli Pang, Tim F. Greten, M. Christine Hollander, Li Yang

**Affiliations:** ^1^ Laboratory of Cancer Biology and Genetics, Center for Cancer Research, National Cancer Institute NIH Bethesda Maryland; ^2^ CCR Collaborative Bioinformatics Resource, Center for Cancer Research, National Cancer Institute NIH Bethesda Maryland; ^3^ Advanced Biomedical Computational Science, Frederick National Laboratory for Cancer Research Sponsored by the National Cancer Institute NIH Frederick Maryland; ^4^ Thoracic and Gastrointestinal Malignancies Branch, Center for Cancer Research, National Cancer Institute NIH Bethesda Maryland

**Keywords:** granulocytic differentiation, immunosuppression, lamin A/C, metastasis, myeloid cells

## Abstract

The immune‐suppressive tumor microenvironment promotes metastatic spread and outgrowth. One of the major contributors is tumor‐associated myeloid cells. However, the molecular mechanisms regulating their differentiation and function are not well understood. Here we report lamin A/C, a nuclear lamina protein associated with chromatin remodeling, was one of the critical regulators in cellular reprogramming of tumor‐associated myeloid cells. Using myeloid‐specific lamin A/C knockout mice and triple‐negative breast cancer (TNBC) mouse models, we discovered that the loss of lamin A/C drives CD11b^+^Ly6G^+^ granulocytic lineage differentiation, alters the production of inflammatory chemokines, decreases host antitumor immunity, and increases metastasis. The underlying mechanisms involve an increased H3K4me3 leading to the upregulation of transcription factors (TFs) Gfi‐1 and C/EBPε. Decreased lamin A/C and increased Gfi‐1 and C/EBPε were also found in the granulocytic subset in the peripheral blood of human cancer patients. Our data provide a mechanistic understanding of myeloid lineage differentiation and function in the immune‐suppressive microenvironment in TNBC metastasis.

**Abbreviations**
CTL: cytotoxic T lymphocyte
LADs: lamina‐associated domains
MDSCs: myeloid‐derived suppressor cells
TNBC: triple‐negative breast cancer

## 1 INTRODUCTION

Distant metastasis is the most common cause of cancer‐associated death.^1^ In particular, the triple‐negative breast cancer (TNBC) subtype has the highest mortality of all breast cancers.^2^, ^3^ Patients with TNBC have no targeted therapy and the current treatments are very inefficient. The understanding and targeting of immune checkpoint inhibitors and recent success using adoptive T‐cell therapy provide encouraging options that could be applicable in targeting TNBC.^4^, ^5^ However, our understanding of the cellular complexity and context‐dependent behaviors of the immune microenvironment is limited in TNBC. Moreover, acquired resistance has been a challenge in achieving therapeutic success.^6^, ^7^

Tumor‐associated chronic inflammation promotes a prometastatic microenvironment where certain immune cells are preferentially recruited and suppress cytotoxic T lymphocyte (CTL)‐mediated antitumor immunity.^8^, ^9^ The majority of prometastatic immune cells are tumor‐associated myeloid cells that show apparent functional plasticity, protumor (M2 or N2) or antitumor (M1 or N1) phenotypes.^10^, ^11^ These include immature myeloid cells or myeloid‐derived suppressor cells (MDSCs), tumor‐associated macrophages, and tumor‐associated neutrophils.^10^, ^11^, ^12^ Increased MDSCs in peripheral blood (PB) and tumor sites are recognized as a poor prognosis factor in patients with several cancer types.^13^, ^14^, ^15^ There are two major subsets of MDSCs, monocytic (CD11b^+^Ly6C^+^) and granulocytic (CD11b^+^Ly6G^+^) MDSCs that have strong tumor‐promoting properties.^16^, ^17^ Myeloid transforming growth factor‐β signaling is critical for the production of cytokines and chemokines that are important in establishing and maintaining the immunosuppressive microenvironment and premetastatic niche.^18^, ^19^

The expansion, differentiation and the functional properties of tumor‐associated myeloid cells are transcriptionally and epigenetically regulated by distinct signals from the metastatic microenvironment.^19^, ^20^, ^21^, ^22^ Chromatin remodeling alters the accessibility of nucleosomal DNA to enhance transcription factor (TF) binding to gene promoters. The physical interactions between chromatin and nuclear structures are critical in this process.^23^, ^24^ In particular, nuclear lamina proteins (ie, lamin A/C and lamin B1) prominently interact with heterochromatin through lamina‐associated domains (LADs) to suppress gene transcription.^25^, ^26^ Interestingly, mutations or protein deficiency of lamin A/C has been shown to affect in vitro differentiation of neutrophils as well as normal hematopoietic stem or progenitor cells through chromatin remodeling.^27^, ^28^ However, the role of lamin A/C in cellular reprogramming of tumor‐associated myeloid cells during metastasis remains to be investigated.

Here, we report that the loss of lamin A/C in myeloid cells enhanced CD11b^+^Ly6G^+^ lineage differentiation, decreased host antitumor immunity, and increased distant metastasis. The underlying mechanisms involve H3K4me3‐mediated upregulation of TFs C/EBPε and Gfi‐1, which are critical for granulocyte lineage differentiation, altered production of inflammatory chemokines, and decreased antigen presentation capacity. These data provide mechanistic insight in understanding the immune microenvironment in TNBC metastasis and point out the systemic correction of immune suppression through epigenetic mechanisms should be considered to enhance the efficacy of cancer immunotherapy.

## 2 MATERIALS AND METHODS

### 2.1 Mice and cell lines

BALB/c, C57Bl/6, and FVB/N mice (female, 6‐ to 8‐week‐old) were purchased from Charles River. The *Lmna* ^flox/flox^ mouse line was obtained from Center for Advanced Preclinical Research at National Cancer Institute (NCI).^29^ *Lmna* ^flox/flox^ mice were bred with LysM‐Cre (B6.129P2‐*Lyz2* ^tm1[cre]Ifo^/J) mice from the Jackson Laboratory to generate the *Lmna* deletion in myeloid cells (*Lmna* ^MyeKO^). MMTV‐Neu and MMTV‐polyoma middle T antigen (PyMT) mice (female, 9‐ to 10‐month‐old) were purchased from the Jackson Laboratory. The Pmel‐1 [B6.Cg‐/Cy Tg(TcraTcrb) 8Rest/J] transgenic mice, specific to human glycoprotein 100 (hgp100) 25 to 33 peptide, were obtained from Dr. Suman K. Vodnala in Surgery Branch, CCR, NCI. All animal protocols were approved by the National Cancer Institute's Animal Care and Use Committee. Murine 4T1 and E0771 cell lines, which are categorized into TNBC, were purchased from the American Type Culture Collection and used for in vivo tumor studies. A highly metastatic E0771 cell line, E0771‐M1, was established from metastatic lung nodules using three sequential rounds of E0771‐M1 tumor transplantation. Cells were cultured in Dulbecco's modified Eagle's medium, supplemented with 10% heat‐inactivated fetal bovine serum, 500 U/mL penicillin and 500 mg/mL streptomycin at 37°C in a humidified atmosphere containing 5% CO_2_ and confirmed to be mycoplasma negative.

### 2.2 Spontaneous or experimental metastasis and in vivo myeloid cell depletion

For orthotopic metastasis, the 3 to 5 × 10^5^ mammary tumor 4T1 or E0771‐M1 cells were injected into the #2 MFP. The number of lung metastases was evaluated after 4 to 5 weeks by Indian ink staining.^19^ For experimental metastasis, the 1 × 10^5^ Lewis lung carcinoma (LLC) cells were injected through the tail vein. The number of metastatic nodules in the lung was evaluated after 2 weeks by Indian ink staining through the trachea.

For in vivo myeloid cell depletion, InVivoMab anti‐mouse Ly6G (1A8; 25 μg/mouse), anti‐mouse Ly6C (Monts; 25 μg/mouse), or IgG2a isotype control (2A3; 25 μg/mouse) were intraperitoneally injected every 2 days starting from day 0 of E0771‐M1 injection until mice were killed.

### 2.3 Ex vivo myeloid cell differentiation or *trans*‐differentiation assay using tumor‐explant supernatant

Approximately, 1 cm^3^ E0771‐M1 tumor was excised and minced into very small pieces under sterile conditions. After transfer into the T75 flask, minced tissues were incubated in 15 mL completed the Rosewell Park Memorial Institute (RPMI)‐1640 medium for 48 hours. Fresh and warmed tumor‐explant supernatant (TES) was used for ex vivo myeloid cell differentiation assay.

For ex vivo myeloid cell differentiation, sorted 5 × 10^5^ hematopoietic stem/progenitor cells (HS/PCs) or myeloid progenitors from bone marrow (BM) were incubated in completed RPMI‐1640 medium with a cocktail of cytokines (20 ng/mL SCF, 20 ng/mL TPO, 20 ng/mL FLT3‐L, and 10 ng/mL IL‐6) for 24 hours. The incubated cells were further cultured in a 12‐well plate with conditioned medium (E0771‐M1 TES:completed PRMI1640 = 1:1) for 72 hours to differentiate into immature myeloid cells. For ex vivo *trans*‐differentiation assay, sorted CD11b^+^Ly6C^+^ and CD11b^+^Ly6G^+^ cells from spleens were directly incubated in 24‐well plate with a conditioned medium for 48 hours. The percentage of myeloid cell subsets was evaluated by flow cytometry analysis.

### 2.4 RNA‐sequencing and chromatin immunoprecipitation‐sequencing analysis

For RNA‐sequencing, the sequencing quality of the 51 to 77 million reads per sample was assessed using FastQC (version 0.11.5), Preseq (version 2.0.3), Picard tools (version 1.119), and RSeQC (version 2.6.4). Reads were then trimmed using Cutadapt (version 1.14) to remove sequencing adapters, before mapping to the mm10 mouse genome using STAR (version 2.5.2b) in two‐pass mode. Overall expression levels were quantified using RSEM version 1.3.0 with GENCODE annotation M12. DESeq2 (version 1.20.0) was used for differential expression analysis. For differential gene expression, *q* ≤ 0.05 and absolute fold change greater than or equal to 1.5 were used to define significant changes.

For the chromatin immunoprecipitation (ChIP)‐sequencing analysis, ChIP‐sequencing data (GSE59636) was downloaded from SRA using SRAtoolkit (version 2.9.2). Sequencing was done on Illumina HiSeq‐1500 and pooled libraries were sequenced at a sequencing depth of ~10 to 15 million aligned reads per sample.

Sequencing quality was assessed using FastQC (version 0.11.5), Preseq (version 2.0.3), Picard tools (version 1.119), and deeptools (version 2.5.0.1). Illumina sequencing adapters were trimmed from reads using cutadapt (version 1.14). Reads were aligned to the mouse genome version mm10 using BWA (version 0.7.15). Duplicate reads of the polymerase chain reaction (PCR) were removed using Picard MarkDuplicates before peak calling. Peaks were called using MACS2 (version 2.1.1) in broad mode. DiffBind (version 2.8.0) and DESeq2 (version 1.20.0) were used for differential binding analysis. Significantly differential bound peaks located within promoter regions were annotated using homer (version 4.10.1). Viewer tracks in WashU Epigenome Browser with Cistrome database were used for comparisons of H3K4me3 or H3K27ac peaks between granulocyte and monocyte. To identify the significant genes targeted by lamin A/C in myeloid cells, differentially expressed genes were first listed up from intersections between lamin A/C‐deficient and wild‐type (wt)‐monocytic subsets (#1), between lamin A/C‐deficient and wt‐granulocytic subsets (#2), and between wt‐monocytic and wt‐granulocytic subsets (#3). Because there were no differences in lamin A/C expression between lamin A/C‐deficient and wt‐granulocytic subsets, genes in the list from intersection #2 were identified as background. Thereafter, overlapped genes of intersection #1 with #3, where the genes from intersection #2 were subtracted, were picked up. All high‐throughput data were deposited into GEO (GSE GSE141124).

### 2.5 RNA interference

ON‐TARGETplus small interfering RNAs (siRNAs) for C/EBPε or Gfi‐1 were purchased from Dharmacon. Transfection of each siRNA into myeloid progenitor cells (5 × 10^4^) was performed using electroporation (mode: Y‐001; Nucleofector II/2b Device) with Amaxa Mouse Macrophage Nucleofector Kit (VPA‐1009; Lonza). The final concentration of the siRNAs was 20 nM. Transduced cells were used for ex vivo differentiation assay.

### 2.6 Mixed Lineage Leukemia 1 inhibitor treatment

MM‐102 (S7265; Selleckchem.com), a selective inhibitor targeting MLL1 that is H3K4 methyltransferase, was diluted with dimethyl sulfoxide (DMSO) to 10 mM and stored in small aliquots at −80°C. Sorted CD11b^+^Ly6C^+^ cells were treated with 25 μM MM‐102 or DMSO control for 24 hours, and total RNA were extracted from treated cells.

### 2.7 Flow cytometry, cell sorting, and interferon‐γ intracellular staining

Single‐cell suspensions were made from spleens, PB, or BM of healthy control or tumor‐bearing mice as well as wt and *Lmna* ^MyeKO^ mice. After hemolysis for 10 minutes at 4°C, cells were labeled with fluorescence‐conjugated antibodies including CD3 (145‐2c11, 1:100; BD Biosciences), CD4 (GK1.5, 1:100; BD Biosciences), CD8 (53‐6.7, 1:100; BD Biosciences), CD19 (1D3, 1:100; BD Biosciences), CD45R/B220 (RA3‐6B2, 1:100; BD Biosciences), CD11b (M1/70, 1:100; BD Biosciences), Ly6C (AL‐21, 1:200; BD Biosciences), Ly6G (1A8, 1:100; BD Biosciences), lineage cocktail (558074; BD Biosciences), CD117 (2B8, 1:100; BD Biosciences), ScaI (E13‐161.7, 1:100; Biolegend), CD34 (RAM34, 1:50; BD Biosciences), Sytox blue nucleic acid stain (S11348, 1:2000; Thermo Fisher Scientific), and 7‐AAD (BD Biosciences), and analyzed on a FACS Calibur, Canto II or LSR II flow cytometer. For sorting, CD3^+^CD4^+^ or CD3^+^CD8^+^ T cells, CD19^+^B220^+^ B cells, Gr‐1^+^CD11b^+^, CD11b^+^Ly6C^+^‐ or CD11b^+^Ly6G^+^‐myeloid cells, or Lin^−^CD117^+^ScaI^−^CD34^+^ progenitor cells were sorted by the FACSAria flow cytometer (BD Biosciences) or magnetically activated cell sorting with Ly6G, and Ly6C microbeads per the manufacturer's protocol (Miltenyi Biotec). HS/PCs were enriched by using immunomagnetic column (Stem Cell Technologies). For human monocytic and granulocytic subsets, PB samples were obtained from patients with advanced gastrointestinal (GI) cancer (n = 8) at the GI Malignancies Section, Medical Oncology Branch at NCI. Written consent was obtained from all patients before blood sampling on a research protocol approved by the NCI Institutional Review Board (NCI‐11‐c‐0112). Cells were enriched by anti‐human CD14 (Miltenyi Biotec), anti‐human CD15 (Miltenyi Biotec), and anti‐HLA‐DR microbeads (Miltenyi Biotec) as previously reported.^19^ For interferon‐γ (IFNγ) intracellular staining of CD3^+^CD8^+^ T cells from metastatic lungs of tumor‐bearing mice, a single‐cell suspension was obtained after lung dissociation with 1 mg/mL collagenase, 120 μg/mL dispase, and 200 μg/mL DNase. The cells were then fixed and permeabilized by a Fixation/Permeabilization kit (BD Biosciences) and stained for IFNγ (XMG1.2, 1:100; BD Biosciences). FlowJo software (Tree Star, Ashland, OR) was used for the analysis of flow cytometry data.

### 2.8 Mouse cytokine antibody array

Mouse cytokine antibody array C1000 (Ray‐biotech) was used to determine the secretory cytokines from monocytic or granulocytic myeloid cells sorted from wt and *Lmna* ^MyeKO^ mice with E0771‐M1 tumor. The whole‐cell lysates (100 μg/sample) from these sorted cells were used for assay and a mouse cytokine antibody array was conducted as per manufacturer's protocol. The amounts of various cytokines were detected using SuperSignal West Dura (Thermo Fisher Scientific) and dot density was determined by using the ImageJ software. Heatmaps were made based on the quantification of dots.

### 2.9 Proliferation and CTL assay of CD8 T cells

For antigen‐specific CD8 T‐cell proliferation regulated by myeloid cells, total splenocytes from Pmel‐1 transgenic mice were labeled with CFSE dye (carboxyfluorescein succinimidyl ester; C34554 1:1000; Thermo Fisher Scientific), which were then cocultured with myeloid cell subsets sorted from spleens of E0771‐M1‐bearing wt and *Lmna* ^Myeko^ mice in culture medium containing 100 IU/mL rIL‐2 and 1 μg/mL hgp100 peptide,^25^, ^26^, ^27^, ^28^, ^29^, ^30^, ^31^, ^32^, ^33^ with a 1:1 ratio (myeloid cells:labeled splenocytes) for 3 days to expand CD8 T cells. The suppressive effect of myeloid cells on CD8 T‐cell proliferation was evaluated by flow cytometry analysis of the CFSE‐labelling intensity of CD8 T cells.

For antigen‐specific cytotoxicity T‐cell activities regulated by myeloid cells, whole splenocytes from Pmel‐1 transgenic mice were cultured in RPMI‐1640 medium containing 100 IU/mL rIL‐2 and 1 μg/mL hgp100 peptide^25^, ^26^, ^27^, ^28^, ^29^, ^30^, ^31^, ^32^, ^33^ for 4 days to expand antigen‐specific CD8 T cells. CFSE (1:1000)‐labeled B16 melanoma cells expressing hgp100 (1 × 10^5^) were cocultured with 2 × 10^5^ expanded CD8 T cells with the addition of myeloid cell subsets sorted from spleens of E0771‐M1‐bearing wt and Lmna^Myeko^ mice at 1:1 ratio (myeloid cells:T cells) for 24 hours. The suppressive effect of myeloid cells on antigen‐specific CTL was evaluated by flow cytometry analysis of dead tumor cells. The percentage of dead CFSE^+^ tumor cells targeted was analyzed by Sytox blue (which labels dead cells).

### 2.10 Isolation of genomic DNA, total RNA, and reverse‐transcription quantitative PCR

Genomic DNA and total RNA was extracted from sorted cells or subsets using ZR‐DUCT DNA/RNA Miniprep kit (Zymo Research) and RNeasy Mini kit (Qiagen), respectively. Complementary DNA (cDNA) was synthesized with a high‐capacity cDNA reverse transcription kit (Applied Biosystems #4368813). Conventional PCR was performed with Taq DNA Polymerase (1 U/μL), dNTPack (Roche) to observe *Lmna* recombination. Reverse‐transcription quantitative PCR (RT‐qPCR) was performed using FastStart Universal SYBR Green Master (Rox) (04913850001; Roche) and ABI 7500 Fast real‐time PCR system (Applied Biosystems). Primers are listed in Table S3. The relative expression level of mRNA transcripts was normalized to that of internal control Glyceraldehyde 3‐phosphate dehydrogenase by using the $2^{-\Delta \Delta C_{t}}$ cycle threshold method.

### 2.11 ChIP‐PCR

ChIP was performed using the ChIP EZ‐Magna ChIP A/G One‐Day Chromatin Immunoprecipitation Kits with the following antibodies against mouse H3K4me3 (Ab8580, 1:1000; Abcam) and normal rabbit immunoglobulin G (IgG) (SC‐2027, 1:1000; Santa Cruz). A total of 3 × 10^6^ sorted cells was fixed and cross‐linked by 1% formaldehyde for precisely 10 minutes with gently shaking (300 rpm). The fixation of chromatin was stopped by 0.125 mol/L glycine. After cell lysis with 200 μL nuclear lysis buffer, sonication was done under 4°C (30 seconds on and 30 seconds off × 15 cycles). After sonication, DNA fragmentation to a length between 200 to 500 base pairs was checked on 1.5% agarose gel. A part of the sheared DNA sample (10%) was used as input. After immunoprecipitation, reverse cross‐links of protein/DNA complexes to free DNA was performed by proteinase K at 62°C. The H3K4me3 binding to a promoter of targeted genes (Cebpe and Gfi‐1) was evaluated by RT‐qPCR using FastStart Universal SYBR Green Master (Rox) (04913850001; Roche) and ABI 7500 Fast real‐time PCR system (Applied Biosystems). Primers are listed in Table S3. Percent input method was used for evaluation: %input = $100\times 2^{\left[(C_{t}^{input}-3.32)-C_{t}^{H3K4me3\,\;or\;\,IgG\,\;control}\right]}$, which were compared between WT and *Lmna* ^Myeko^ mice.

### 2.12 Western blot analysis

Protein extracts from Gr‐1^+^CD11b^+^, CD11b^+^Ly6C^+^, and CD11b^+^Ly6G^+^ cells were analyzed by Western blot analysis. The following primary antibodies were used: lamin A/C (SC‐6214, 1:1000; Santa Cruz), Gfi‐1 (ab21061, 1:2000; Abcam), C/EBPε (NBP1‐85446, 1:1000; Novus) Acetyl‐H3 (9649S, 1:1000; CST), Histone 3 (14269S, 1:1000; CST), and β‐actin (SC69879, 1:2000; Santa Cruz). Anti‐mouse/rabbit/goat secondary antibodies were purchased from Bio‐Rad (1:3000‐5000 dilution). The blotting images were taken by ChemiDoc Touch Imaging System (Bio‐Rad), and Image Lab software (Bio‐Rad) was used for analysis.

### 2.13 Immunofluorescence

Cells were fixed in 100% cold methanol for 10 minutes and permeabilized for 10 minutes in 0.3% Triton X‐100 in phosphate‐buffered saline. After blocking with 5% normal Donkey serum (Ab7475; Abcam) for 1 hour, cells were incubated overnight at 4°C with following primary antibodies: lamin A/C (sc6215, 1:100; Santa Cruz), lamin B1 (ab16048, 1:100; Abcam 1:1000), H3K4me3 (ab8580, 1:500; Abcam), and H3K9me3 (ab8898, 1:500; Abcam). The cells were then incubated with Alexa Fluor 488 and 594 donkey anti‐goat/rabbit IgG (H+L) (A‐11055/R‐37119, Life Technologies 1:500) for 2 hours, and then mounted with Antifade Mounting medium with DAPI (4′,6‐diamidino‐2‐phenylindole) (VECTASHIELD). Fluorescence was examined by microscopy (Olympus IX‐81) or confocal laser scanning microscope (LSM710; Zeiss). The fluorescence intensity of lamin A/C in each cell was measured by the ImageJ software.

### 2.14 Human correlative studies

Publicly available datasets of human peripheral mononuclear cells (PBMCs) from patients with breast cancer (GSE27567) was used to investigate the correlation of *Lmna*‐targeted gene expression levels. GEO2R analyzer was used for collecting expression values of samples in each data set. The logarithm with base 2 (expression value/median value) was calculated in each sample.

### 2.15 Statistical analysis

GraphPad Prism was used for graphs and for statistics. Unless otherwise indicated, all data were analyzed using the one‐tailed Student *t* test and are expressed as mean ± standard error of the mean. Differences were considered statistically significant when the *p*‐value was <.05.

## 3 RESULTS

### 3.1 Lamin A/C expression in granulocytic or monocytic myeloid cell differentiation

**Figure 1:**
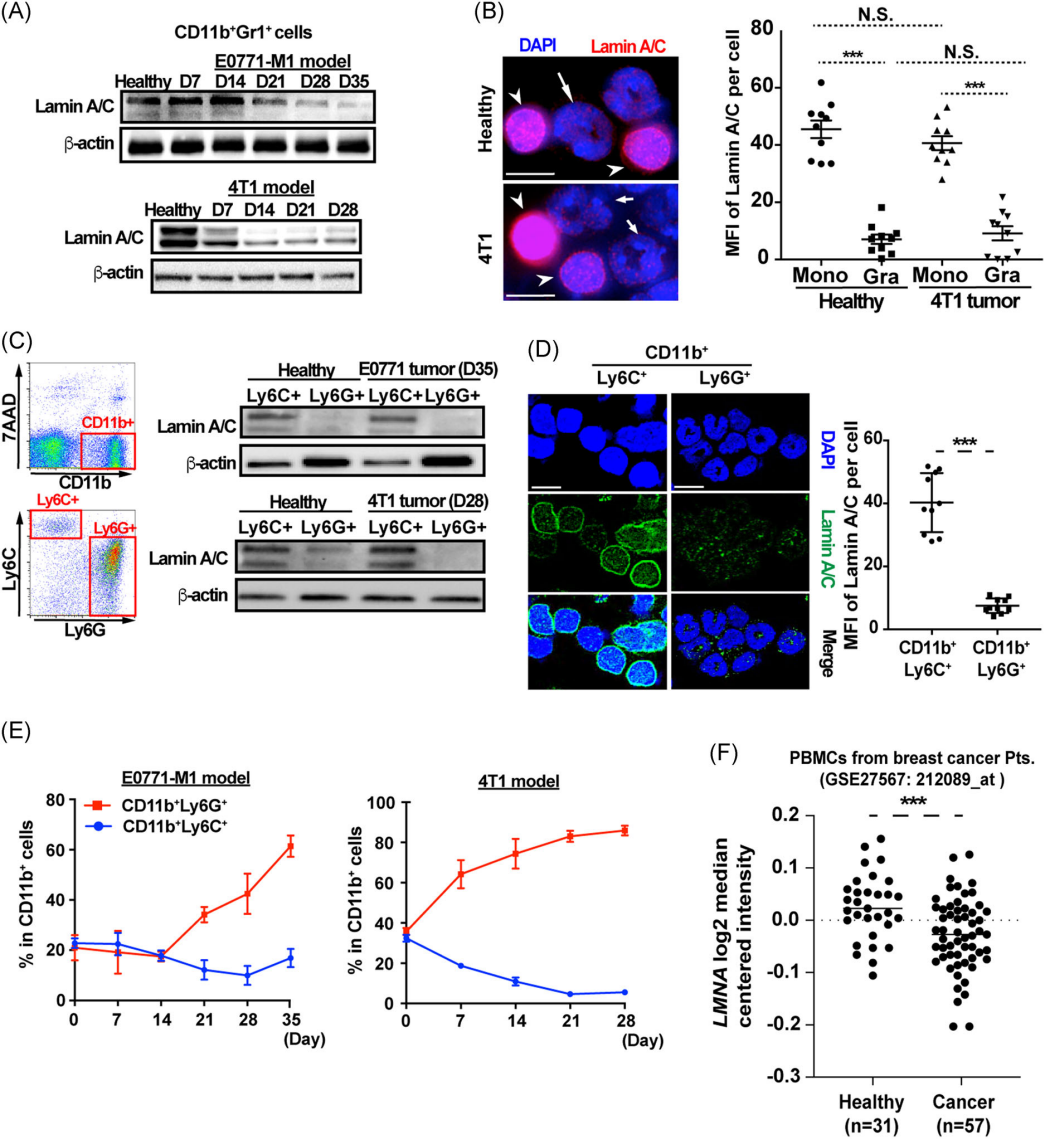
Lamin A/C expression is decreased in granulocytic myeloid cells. A, Lamin A/C Western blot analysis of CD11b^+^Gr1^+^cells from spleens of E0771‐M1 (upper) or 4T1 (lower) tumor‐bearing mice. B, Immunofluorescence (IF) of lamin A/C (red) and DAPI (blue) in splenic Gr1^+^CD11b^+^ cells of healthy (upper) and 4T1 tumor‐bearing mice (lower). Monocytic (arrow heads) and granulocytic (arrows) are indicated. The quantitative data of lamin A/C mean fluorescence intensity per cell is on the right (n = 10 cells evaluated). C, Lamin A/C expression in myeloid cell subsets: Left: gating strategy. Right: lamin A/C Western of splenic CD11b^+^Ly6C^+^ and CD11b^+^Ly6G^+^ cells from healthy control and E0771‐M1 (upper right) or 4T1 (lower right) tumor‐bearing mice. D, Lamin A/C IF (green) and DAPI (blue) in sorted CD11b^+^Ly6C^+^ and CD11b^+^Ly6G^+^cells from spleens of 4T1 tumor‐bearing mice (left). The quantitative data of lamin A/C mean fluorescence intensity per cell is on the right (n = 10 cells evaluated). E, Percentage of CD11b^+^Ly6G^+^ granulocytic subset and the CD11b^+^Ly6C^+^ monocytic subset during metastatic progression in E0771‐M1 and 4T1 tumor models. F, Lamin A/C expression levels in human peripheral blood mononuclear cells (PBMCs) from breast cancer patients (n = 57) and healthy donors (n = 31) (GSE27567). Data are represented as mean ± standard error of the mean. All scale bars = 10 μm. DAPI, 4′,6‐diamidino‐2‐phenylindole; *NS*, not significant. *P < .05; ***P < .001 [Color figure can be viewed at wileyonlinelibrary.com]

We first noticed a gradual decrease of lamin A/C in immature myeloid cells or Gr‐1^+^CD11b^+^ cells during mammary tumor progression in both E0771‐M1‐ and 4T1 tumor models (Figure 1A). This result was also observed in genetically engineered MMTV‐PyMT and MMTV‐Neu transgenic mice (Figure S1A). Gr‐1^+^CD11b^+^ cells are composed of monocytic and granulocytic myeloid subsets.^17^ The granulocytic subset showed a clear lack of lamin A/C expression compared with that of a monocytic subset (Figure 1B), with no difference in lamin B1 expression (Figure S1B). This difference in lamin A/C expression between the two myeloid subsets was further verified in sorted CD11b^+^Ly6G^+^ cells from healthy control and E0771‐M1 or 4T1 tumor‐bearing mice (Figures 1C,D and S1C). Notably, the CD11b^+^Ly6G^+^ granulocytic subset but not the CD11b^+^Ly6C^+^ monocytic subset was expanded during metastatic progression in both E0771‐M1 and 4T1 tumor models (Figure 1E). Thus, we speculate that lamin A/C may have a role in myeloid cell lineage differentiation under tumor conditions. In addition, lamin A/C expression was decreased in PBMCs from breast cancer patients compared to those from healthy donors (Figure 1F; GSE27567 data set).

### 3.2 Myeloid‐specific lamin A/C knockout promotes a granulocytic lineage differentiation that enhances tumor metastasis

**Figure 2:**
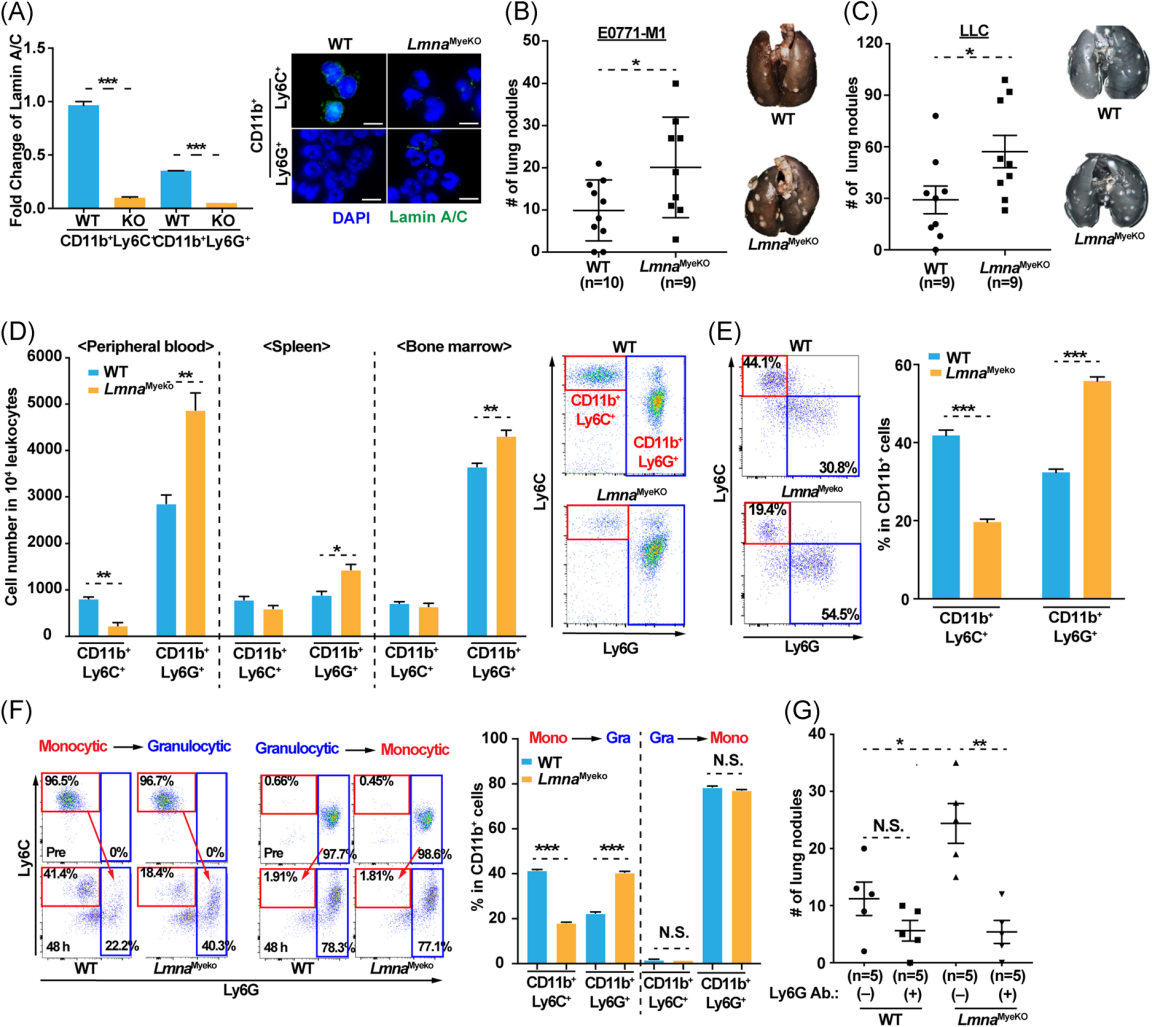
Myeloid‐specific deletion of lamin A/C decreases lung metastasis through the correction of myeloid lineage differentiation. A, Percentage of splenic CD11b^+^Ly6C^+^ cells (blue) and CD11b^+^Ly6G^+^cells (red) in time‐course experiments for E0771‐M1 (left) or 4T1 (right) tumor‐bearing mice at indicated days after tumor injection. Left: RT‐qPCR for fold change of lamin A/C and Right: lamin A/C IF (green) and DAPI (blue) in CD11b^+^Ly6C^+^ and CD11b^+^Ly6G^+^ cells from the spleen of E0771‐M1 tumor‐bearing wt or *Lmna* ^MyeKO^ mice. B, C, Left: the number of metastatic nodules in the lungs of wt and *Lmna* ^MyeKO^ mice with E0771‐M1 breast tumor (n = 9‐10, B) or the LLC experimental metastasis (n = 9, C). Representative lungs stained by Indian ink on the right panels. D, The number of CD11b^+^Ly6C^+^ and CD11b^+^Ly6G^+^ cells in 10^4^ leukocytes of peripheral blood, spleen, and bone marrow of E0771‐M1 tumor‐bearing wt mice or *Lmna* ^MyeKO^ mice. Representative flow cytometry analysis for peripheral blood samples is shown on the right. E, Ex vivo differentiation of hematopoietic stem/progenitor cells (HS/PCs) from wt or *Lmna* ^MyeKO^ mice bearing E0771‐M1 tumors. Left: representative flow cytometry analysis. Right: percentage of CD11b^+^Ly6C^+^ and CD11b^+^Ly6G^+^ cells. F, Ex vivo *trans*‐differentiation of CD11b^+^Ly6C^+^ and CD11b^+^Ly6G^+^ cells from wt or *Lmna* ^MyeKO^ mice bearing E0771‐M1 tumors. Left panels: flow cytometry analysis of Ly6C^+^ monocytic and Ly6G^+^ granulocytic subsets. Arrows indicate myeloid *trans*‐differentiation. Right: percentage of CD11b^+^Ly6C^+^ cells and CD11b^+^Ly6G^+^ cells. G, The number of lung nodules in E0771‐M1 tumor‐bearing wt and *Lmna* ^MyeKO^ mice (n = 5) with or without in vivo depletion of CD11b^+^Ly6G^+^ cells. The data are represented as mean ± standard error of the mean. DAPI, 4′,6‐diamidino‐2‐phenylindole; IF, immunofluorescence; KO, knockout; LLC, Lewis lung carcinoma; RT‐qPCR, reverse‐transcription quantitative polymerase chain reaction; wt, wild‐type. *P < .05; **P < .01; ***P < .001 [Color figure can be viewed at wileyonlinelibrary.com]

Lamin A/C has been shown to modulate human hematopoietic differentiation programs.^28^ To examine whether lamin A/C has any effect on myeloid lineage differentiation, mice with *Lmna* gene deletion specifically in myeloid cells (*Lmna* ^MyeKO^) were generated through the cross‐breeding of *Lmna* floxed mice with LysM‐Cre transgenic mice (Figure S2A). Gr‐1^+^CD11b^+^ myeloid cells, but not CD19^+^B220^+^ B cells or CD3^+^ T cells, showed lamin A/C downregulation in *Lmna* ^MyeKO^ mice (Figure S2B). Within the myeloid compartment, *Lmna* recombination was observed in both granulocytic and monocytic subsets, which was validated by RT‐qPCR and immunofluorescence analysis (Figures 2A and S2C). Interestingly, *Lmn*a^MyeKO^ mice bearing E0771‐M1, a TNBC breast cancer cell line variant from E0771 with high metastatic capacity, showed an increase in metastatic lung nodules but with no effect on primary tumor size when compared with wt mice (Figures 2B and S2D). A similar result was observed in the experimental metastasis of LLC cells (Figure 2C). These results suggest that lamin A/C has critical functions in suppressing cancer metastasis.

There was no difference in the number of CD3^+^CD4^+^ T cells, CD3^+^CD8^+^ T cells, and CD19^+^B220^+^ B cells comparing E0771‐M1 tumor‐bearing wt with *Lmna* ^MyeKO^ mice (Figure S2E). However, *Lmna* ^MyeKO^ mice bearing E0771‐M1 tumors showed an increased number of CD11b^+^Ly6G^+^ cells in PB, spleen, and BM when compared to those from wt mice, while the CD11b^+^Ly6C^+^ cells were decreased in PB but no changes in the spleen or BM (Figure 2D). Consistently, HS/PCs from *Lmna* ^MyeKO^ mice showed enhanced differentiation into CD11b^+^Ly6G^+^ cells in E0771‐M1 tumor‐conditioned medium in ex vivo culture (Figures 2E and S2F). Moreover, lamin A/C knockout enhanced *trans*‐differentiation to granulocytic CD11b^+^Ly6G^+^ cells in ex vivo culture compared to those from wt mice (Figures 2F and S2G). In contrast, CD11b^+^Ly6G^+^ cells were not able to *trans*‐differentiate into CD11b^+^Ly6C^+^ cells (Figure 2F). These data suggest that loss of lamin A/C promotes and accumulates granulocytic lineage differentiation in tumor‐bearing mice. To examine whether the increased granulocytic subset due to loss of lamin A/C is responsible for enhanced lung metastasis, CD11b^+^Ly6G^+^ cells were depleted with Ly6G neutralizing antibody (Figure S2H), which showed a decreased number of metastatic nodules in E0771‐M1 tumor‐bearing *Lmna* ^MyeKO^ mice (Figure 2G). In contrast, depletion of CD11b^+^Ly6C^+^ cells did not significantly affect metastatic phenotype in E0771‐M1 tumor‐bearing *Lmna* ^MyeKO^ mice (Figure S2I). These data demonstrate that myeloid‐specific lamin A/C knockout increases lung metastasis via increased granulocytic myeloid cells.

### 3.3 Loss of lamin A/C elicits H3K4me3‐mediated enhancing the expression of transcription factors enabling granulocytic cell differentiation

**Figure 3:**
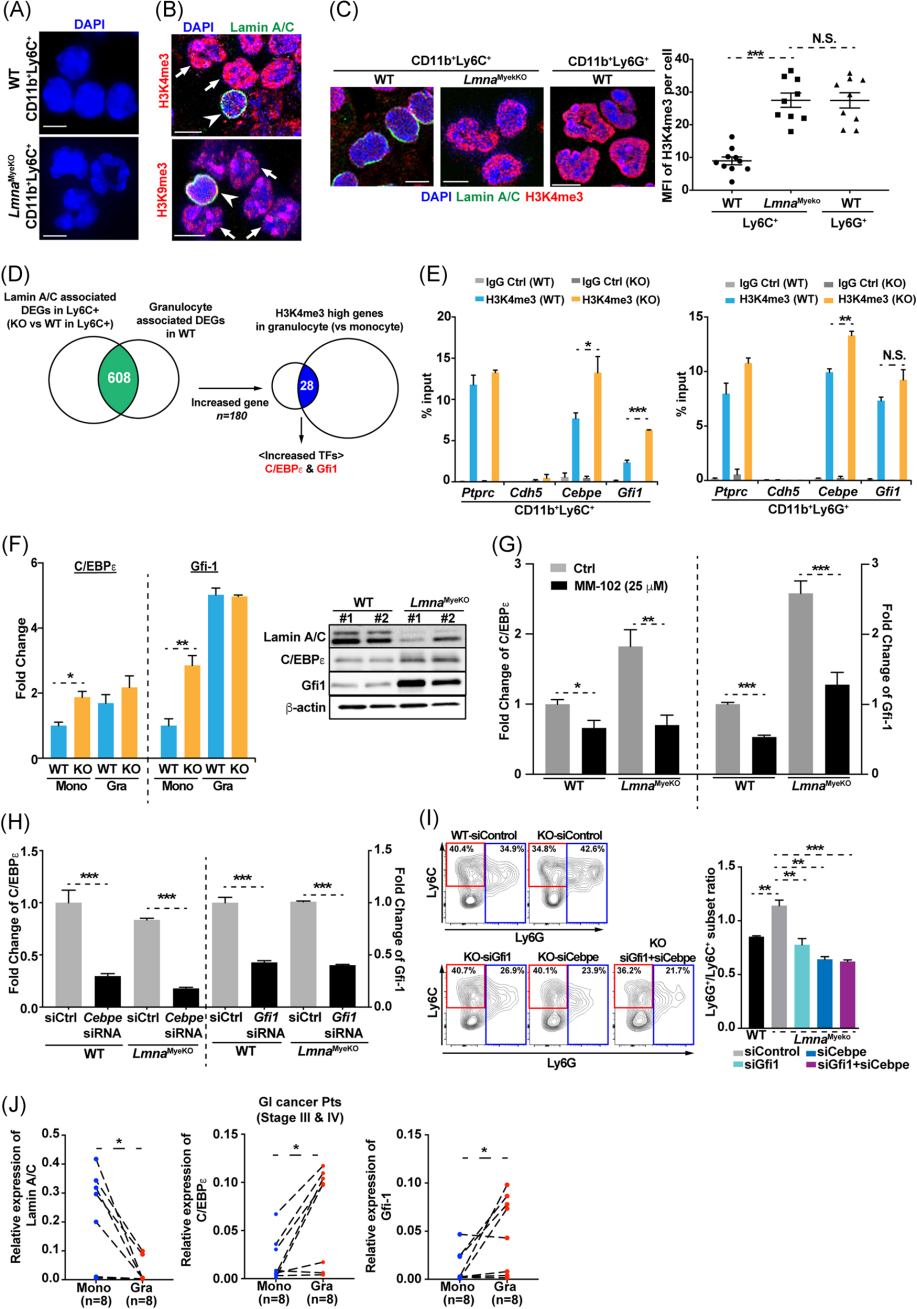
Deletion of lamin A/C promotes H3K4me3‐mediated epigenetic activation and granulocytic lineage differentiation. A, Hyperlobular nuclear morphology in lamin A/C‐deficient CD11b^+^Ly6C^+^ cells sorted from spleens of E0771‐M1 tumor‐bearing *Lmna* ^MyeKO^ compared to the wt mice. B, Lamin A/C IF (green), H3K4me3 or H3K9me3 (red) and DAPI (blue) in a monocytic subset (arrow heads) and a granulocytic subset (arrows) from 4T1 tumor‐bearing mice. C, Left: lamin A/C IF (green), H3K4me3 (red), and DAPI (blue) in CD11b^+^Ly6C^+^ cells from spleens of E0771‐M1 tumor‐bearing wt and *Lmna* ^MyeKO^ mice. CD11b^+^Ly6G^+^cells from wt mice as positive control for H3K4me3. Right: the quantitative data of H3K4me3 mean fluorescence intensity per cell (n = 9‐10 cells evaluated). D, Schematic identification of key TFs C/EBPε and Gfi‐1 (in red). Transcriptome from CD11b^+^Ly6C^+^ and CD11b^+^Ly6G^+^ cells of wt and *Lmna* ^MyeKO^ mice was intersected with the public H3K4me3‐ChIP‐seq data set. Green shows the differential expressed genes comparing lamin A/C deficient with wt‐CD11b^+^Ly6C^+^ cells. E, H3K4me3‐ChIP‐PCR for C/EBPε or Gfi‐1 expression. *Ptprc* and *Cdh5* as positive and negative controls respectively. F, C/EBPε or Gfi‐1 RT‐qPCR (left) and Western blot analysis (right). E, F, Both myeloid subsets were sorted from spleens of wt and *Lmna* ^MyeKO^ mice. G, C/EBPε or Gfi‐1 RT‐qPCR of sorted CD11b^+^Ly6C^+^ cells treated with MLL1 inhibitor MM‐102. H, C/EBPε or Gfi‐1 RT‐qPCR of sorted Lin^–^CD117^+^ScaI^–^CD34^+^ progenitor cells from E0771‐M1 tumor‐bearing wt and *Lmna* ^MyeKO^ mice with siRNA knockdown of C/EBPε or Gfi‐1. I, Ex vivo myeloid lineage differentiation from progenitor cells of wt and *Lmna* ^MyeKO^ mice. Upper: representative flow cytometry of CD11b^+^Ly6C^+^ and CD11b^+^Ly6G^+^ cells. Lower: quantitative data. J, RT‐qPCR of lamin A/C (left), C/EBPε (middle), and Gfi‐1 (right) in monocytic (Mono) and granulocytic (Gra) subsets in peripheral blood from advanced GI cancer patients (n = 8). The data are represented as mean ± standard error of the mean. All Scale bars = 10 μm. ChIP, chromatin immunoprecipitation; DAPI, 4′,6‐diamidino‐2‐phenylindole; IF, immunofluorescence; IgG, immunoglobulin G; KO, knockout; MLL1, mixed lineage leukemia 1; NS, not significant. RT‐qPCR, reverse‐transcription quantitative polymerase chain reaction; wt, wild‐type. *P < .05, **P < .01, ***P < .001 [Color figure can be viewed at wileyonlinelibrary.com]

Lamin A/C prominently interacts with heterochromatin through LADs to suppress gene transcription.^25^, ^26^ To dissect the molecular mechanisms in the enhanced granulocytic lineage differentiation in *Lmna* ^MyeKO^ mice, we first noticed the deformed and partially segmented nuclei in CD11b^+^Ly6C^+^ cells similar to that CD11b^+^Ly6G^+^ cells (Figure 3A). This led us to investigate whether loss of lamin A/C causes chromatin remodeling, and changes in H3K4me3 and H3K9me3, markers of euchromatin and heterochromatin, respectively. H3K4me3 was visibly higher in Gr1^+^CD11b^+^ cells expressing low levels of lamin A/C compared with those expressing high levels of lamin A/C (Figure 3B). H3K4m3‐high and lamin A/C‐low cells also showed hyperlobular nuclear shape (Figure 3B). In contrast, there was no difference in H3K9me3 (Figure 3B). The higher H3K4me3 level was further validated in lamin A/C‐deficient CD11b^+^Ly6C^+^ cells compared with wt control, which is comparable to that of wt‐CD11b^+^Ly6G^+^ cells showing decreased lamin A/C (Figure 3C). These data indicate that loss of lamin A/C causes epigenetic activation in nuclei of immature myeloid cells.

RNA‐seq for monocytic and granulocytic myeloid cells from wt and *Lmna* ^MyeKO^ mice revealed 608 differentially expressed genes associated with the presence of lamin A/C (Figures 3D and S3A). Among those, 180 genes were upregulated in lamin A/C‐deficient CD11b^+^Ly6C^+^ cells (Table S1). Analysis of public datasets of H3K4me3‐ChIP‐seq for monocytes or granulocytes (Cistrome database) showed increased H3K4me3 peaks at promoter regions of monocytic markers (eg Csf1r, Ccr2, and Ly6C) or granulocytic markers (eg Ly6G, S100a8, and S100a9), respectively (Figure S3B). The intersection of H3K4me3 peaks with 180 increased genes in lamin A/C‐deficient CD11b^+^Ly6C^+^ cells revealed 28 genes (Figure 3D and Table S2). Among them, there were two TFs, growth factor independent 1 (Gfi‐1) and CCAAT/enhancer‐binding protein (C/EBPε), which are critical for granulocytic lineage differentiation (Figure 3D). Upregulation of these TFs was confirmed by H3K4me3‐ChIP‐seq analysis (Cistrome database) and ChIP‐PCR, RT‐qPCR, and Western blot analysis (Figures 3E,F and S3C). When treated with an inhibitor of MLL1, a writer for H3K4me3, the increased expressions of C/EBPε and Gfi‐1 in the lamin A/C‐deficient CD11b^+^Ly6C^+^ cells were minimized (Figure 3G). C/EBPε and Gfi‐1knock down by siRNA in lamin A/C‐deficient Lin^−^CD117^+^ScaI^−^CD34^+^ myeloid progenitors diminished the enhanced granulocytic lineage differentiation (Figures 3H,I and S3D,S3E). Together these data suggest that loss of lamin A/C elicits H3K4me3‐mediated upregulation of C/EBPε and Gfi‐1 that promote a granulocytic lineage differentiation. For human studies, monocytic (CD11b^+^CD14^+^HLA‐DR^−^) and granulocytic (CD11b^+^CD14^−^CD15^+^) subsets from PB of advanced GI cancer patients were isolated and evaluated. RT‐qPCR analyses revealed a lower level of lamin A/C expression in the granulocytic subset than that in the monocytic subset, and the lamin A/C expression was inversely correlated with C/EBPε and Gfi‐1 levels (Figure 3J), which is consistent with the observation from mouse models, suggesting the correlation of decreased lamin A/C with increased C/EBPε and Gfi‐1.

### 3.4 Loss of lamin A/C increased immune‐suppressive function of CD11b^+^Ly6C^+^ cells

**Figure 4:**
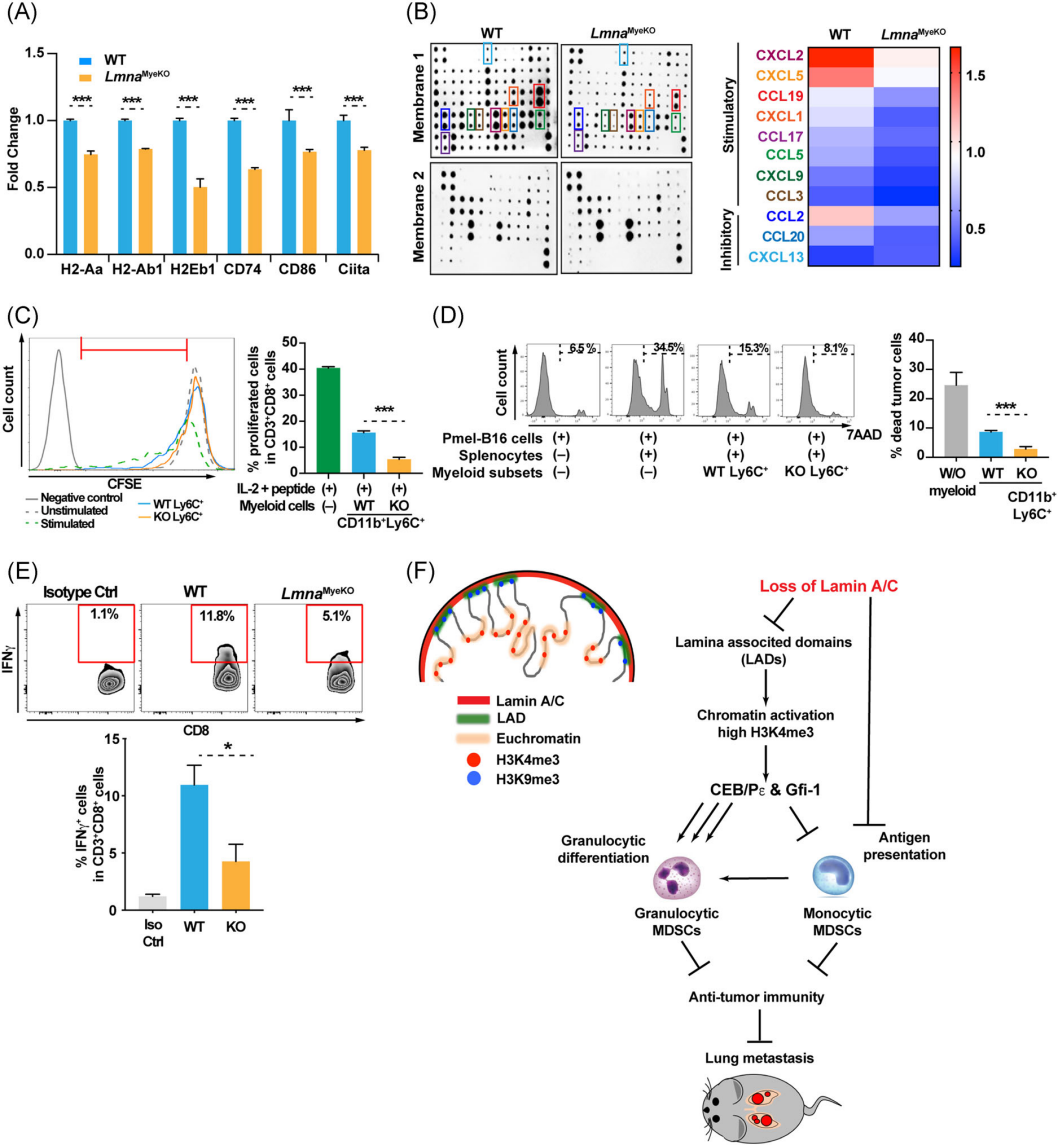
Lamin A/C deletion suppresses antigen‐presenting function and hosts antitumor immunity. A, RT‐qPCR validation of genes targeted by lamin A/C in CD11b^+^Ly6C^+^ cells from spleens of E0771‐M1 tumor‐bearing wt and *Lmna* ^MyeKO^ mice (n = 3). B, Cytokine protein array (left), and Heatmap of stimulatory and inhibitory chemokines (right) of CD11b^+^Ly6C^+^ cells from E0771‐M1 tumor‐bearing wt and *Lmna* ^MyeKO^ mice. C, CD8 T‐cell proliferation in coculture with sorted CD11b^+^Ly6C^+^ cells (Ly6C^+^) from spleens of E0771‐M1 tumor‐bearing wt and *Lmna* ^MyeKO^ mice (n = 3) (left), with quantitative data (right). D, CTL assays: flow cytometry of CFSE dye‐labeled B16 tumor cells expressing gp100 (Pmel) in coculture with splenocytes from TCR‐Pmel‐transgenic mice, with or without the addition of sorted CD11b^+^Ly6C^+^ cells (Ly6C^+^) from spleens of E0771‐M1 tumor‐bearing wt and *Lmna* ^MyeKO^ mice. E, Flow cytometry of IFNγ^+^CD8^+^ T cells in metastatic lungs from E0771‐M1 tumor‐bearing wt and *Lmna* ^MyeKO^ mice (n = 3). F, Schematic hypothesis of lamin A/C functions in myeloid cell differentiation and host antitumor immunity. Quantitative data on the right. All data are represented as mean ± standard error of the mean. CFSE, carboxyfluorescein succinimidyl ester; CTL, cytotoxic T lymphocyte; IFNγ, interferon‐γ; KO, knockout; RT‐qPCR, reverse‐transcription quantitative polymerase chain reaction; TCR, T‐cell receptor; wt, wild‐type. *P < .05, ***P < .001 [Color figure can be viewed at wileyonlinelibrary.com]

RNA‐seq analysis indicated that loss of lamin A/C decreased the expression of genes that are critical for antigen‐presenting pathways such as MHC (major histocompatibility complex) class II (H2‐Aa, H2‐Ab1, and H2‐Eb1), CD74, CD86, and Ciita in CD11b^+^Ly6C^+^ cells, which was confirmed by RT‐qPCR (Figure 4A). The monocytic myeloid cell subset is already known to be immunosuppressive.^30^, ^31^ Loss of lamin A/C apparently further decreased the antigen‐presenting capacity of these monocytic myeloid cells (Figure 4A). Cytokine protein array analysis showed a decreased immune‐stimulatory chemokine profile in lamin A/C‐deficient CD11b^+^Ly6C^+^ cells compared to wt control (Figure 4B). This is likely critical in trafficking of antigen‐presenting cells into lymph node and cancer tissues as antigen presentation to effector cells is strictly regulated by secretory cytokines and chemokines.^32^ Unexpectedly, there was no obvious difference in M1/M2 cytokine profile between wt and lamin A/C‐deficient CD11b^+^Ly6C^+^ cells as both IL‐12 (M1 cytokine) and IL‐4 (M2 cytokine) were decreased in lamin A/C‐deficient cells (Figure S4A). In functional assays, lamin A/C‐deficient CD11b^+^Ly6C^+^ cells inhibited CD8 T‐cell proliferation (Figure 4C) and antigen‐specific cytotoxicity of CTLs (Figure 4D). Consistently, IFNγ^+^CD8^+^ Τ cells were also decreased in metastatic lungs of E0771‐M1 tumor‐bearing *Lmna* ^MyeKO^ mice compared with wt control (Figure 4E and S4B). These data suggest that loss of lamin A/C not only promotes the granulocytic lineage differentiation but also negatively regulates monocytic myeloid cell function, leading to attenuating antitumor immunity (Figure 4F).

## 4 DISCUSSION

We report for the first time that myeloid‐specific lamin A/C attenuation promotes tumor metastasis through increased granulocytic lineage differentiation and decreased CTL‐mediated antitumor immunity. These studies demonstrate the importance of lamin A/C–H3K4me3‐mediated epigenetic regulation of C/EBPε and Gfi‐1 in myeloid lineage differentiation, function as well as host antitumor immunity.

Our data provide insight to the molecular mechanisms of well‐noted immune suppression, dysregulation of myelopoiesis under tumor conditions.^22^, ^30^, ^31^ Previous report showed a role of lamin B receptor in all *trans*‐retinoic acid induced granulopoiesis, which was accompanied by downregulation of lamin A/C.^33^ In addition, overexpression of lamin A/C impaired nucleus shape transition which is important for granulopoiesis.^34^ However, the detailed mechanism of loss of lamin A/C in granulocytic differentiation was largely unknown. In our studies, loss of lamin A/C is a causal factor in driving granulocytic lineage differentiation. Lamin A/C is a nuclear lamina component that retains nuclear shape and makes physical connections with chromatin and loss of lamin A/C causes chromatin remodeling and altered gene transcription profiles.^25^, ^26^ Intersection of the transcriptome from Lmna‐deficient myeloid cell subsets with public H3K4me3‐ChIP‐seq^35^ provided creditable profiles of genes that were epigenetically regulated by lamin A/C. We found lamin A/C is associated with chromatin remodeling in myeloid cells through H3K4me3‐mediated expression of C/EBPε and Gfi‐1 which are important in granulocytic lineage differentiation of immature myeloid cells.^36^, ^37^ In fact, the abnormal differentiation of HS/PCs in BM leads to an expansion of immune‐suppressive granulocytic subset by several transcrption factors, such as STAT3, IRF8, C/EBPβ, and RB1.^38^ We identify two TFs C/EBPε and Gfi‐1 in promoting granulocytic lineage differentiation and in driving a prometastatic microenvironment.

In addition to the granulocytic lineage differentiation, we revealed that loss of lamin A/C also contributes to decreased immune surveillance. Lamin A/C deficiency affects two major aspects of host antitumor immunity: attenuation of the antigen‐presenting pathway and decreased chemoattractant for interaction with effector T cells. For the first aspect, the monocytic subset under tumor condition shows impaired antigen presentation which is consistent with published reports.^30^, ^31^ Loss of lamin A/C negatively regulates monocytic myeloid cell function, leading to attenuated antitumor immunity. The second aspect is particularly interesting. Our data suggest that the critical chemokines that bring the APC and effector T cells into approximate interactions are at stake when lamin A/C expression is reduced (Figure 4B). Our observations are supported by previous studies reporting that lamina‐associated domains contain genes important in host immune response and loss of lamin‐mediated chromatin remodeling affects the regulation of those genes.^26^

In conclusion, our studies provide mechanistic insight for lamin A/C mediated epigenetic regulation of myeloid lineage differentiation and immune‐suppressive function, suggesting that proper epigenetic inhibitors could be utilized to redirect myeloid lineage differentiation and to enhance host antitumor immunity. This systemic correction of the immune‐suppressive microenvironment provides additional options to enhance the efficacy of cancer immunotherapy.

## CONFLICT OF INTERESTS

The authors declare that there are no conflict of interests.

## Supporting information

Supporting information

Supporting information

Supporting information

Supporting information

Supporting information

Supporting information

Supporting information

## References

[1] Steeg PS . Targeting metastasis. Nat Rev Cancer. 2016;16(4):201‐218. 27009393 10.1038/nrc.2016.25 PMC7055530

[2] Liedtke C , Mazouni C , Hess KR , et al. Response to neoadjuvant therapy and long‐term survival in patients with triple‐negative breast cancer. J Clin Oncol. 2008;26(8):1275‐1281. 18250347 10.1200/JCO.2007.14.4147

[3] Foulkes WD , Smith IE , Reis‐Filho JS . Triple‐negative breast cancer. N Engl J Med. 2010;363(20):1938‐1948. 21067385 10.1056/NEJMra1001389

[4] Bianchini G , Balko JM , Mayer IA , Sanders ME , Gianni L . Triple‐negative breast cancer: challenges and opportunities of a heterogeneous disease. Nat Rev Clin Oncol. 2016;13(11):674‐690. 27184417 10.1038/nrclinonc.2016.66 PMC5461122

[5] Zacharakis N , Chinnasamy H , Black M , et al. Immune recognition of somatic mutations leading to complete durable regression in metastatic breast cancer. Nat Med. 2018;24(6):724‐730. 29867227 10.1038/s41591-018-0040-8 PMC6348479

[6] Zaretsky JM , Garcia‐Diaz A , Shin DS , et al. Mutations associated with acquired resistance to PD‐1 blockade in melanoma. N Engl J Med. 2016;375(9):819‐829. 27433843 10.1056/NEJMoa1604958 PMC5007206

[7] Restifo NP , Smyth MJ , Snyder A . Acquired resistance to immunotherapy and future challenges. Nat Rev Cancer. 2016;16(2):121‐126. 26822578 10.1038/nrc.2016.2 PMC6330026

[8] Coussens LM , Werb Z . Inflammation and cancer. Nature. 2002;420(6917):860‐867. 12490959 10.1038/nature01322 PMC2803035

[9] Grivennikov SI , Greten FR , Karin M . Immunity, inflammation, and cancer. Cell. 2010;140(6):883‐899. 20303878 10.1016/j.cell.2010.01.025 PMC2866629

[10] Noy R , Pollard JW . Tumor‐associated macrophages: from mechanisms to therapy. Immunity. 2014;41(1):49‐61. 25035953 10.1016/j.immuni.2014.06.010 PMC4137410

[11] Fridlender ZG , Sun J , Kim S , et al. Polarization of tumor‐associated neutrophil phenotype by TGF‐beta: “N1” versus “N2” TAN. Cancer Cell. 2009;16(3):183‐194. 19732719 10.1016/j.ccr.2009.06.017 PMC2754404

[12] Gabrilovich DI , Nagaraj S . Myeloid‐derived suppressor cells as regulators of the immune system. Nat Rev Immunol. 2009;9(3):162‐174. 19197294 10.1038/nri2506 PMC2828349

[13] Mirza N , Fishman M , Fricke I , et al. All‐trans‐retinoic acid improves differentiation of myeloid cells and immune response in cancer patients. Cancer Res. 2006;66(18):9299‐9307. 16982775 10.1158/0008-5472.CAN-06-1690 PMC1586106

[14] Jordan KR , Amaria RN , Ramirez O , et al. Myeloid‐derived suppressor cells are associated with disease progression and decreased overall survival in advanced‐stage melanoma patients. Cancer Immunol Immunother. 2013;62(11):1711‐1722. 24072401 10.1007/s00262-013-1475-x PMC4176615

[15] Roberts ME , Wagner L , Zorjan S , Nemeth E , van Toor D , Czaplinski M . Testing the Situationism Scale in Europe: scale validation, self‐regulation and regional differences. Int J Psychol. 2017;52(4):264‐272. 28703327 10.1002/ijop.12211

[16] Marvel D , Gabrilovich DI . Myeloid‐derived suppressor cells in the tumor microenvironment: expect the unexpected. J Clin Invest. 2015;125(9):3356‐3364. 26168215 10.1172/JCI80005 PMC4588239

[17] Bronte V , Brandau S , Chen SH , et al. Recommendations for myeloid‐derived suppressor cell nomenclature and characterization standards. Nat Commun. 2016;7:12150. 27381735 10.1038/ncomms12150 PMC4935811

[18] Pang Y , Gara SK , Achyut BR , et al. TGF‐beta signaling in myeloid cells is required for tumor metastasis. Cancer Discov. 2013;3(8):936‐951. 23661553 10.1158/2159-8290.CD-12-0527 PMC4678771

[19] Ishii H , Vodnala SK , Achyut BR , et al. miR‐130a and miR‐145 reprogram Gr‐1(+)CD11b(+) myeloid cells and inhibit tumor metastasis through improved host immunity. Nat Commun. 2018;9(1):2611. 29973593 10.1038/s41467-018-05023-9 PMC6031699

[20] Ji H , Ehrlich LIR , Seita J , et al. Comprehensive methylome map of lineage commitment from haematopoietic progenitors. Nature. 2010;467(7313):338‐342. 20720541 10.1038/nature09367 PMC2956609

[21] Alvarez‐Errico D , Vento‐Tormo R , Sieweke M , Ballestar E . Epigenetic control of myeloid cell differentiation, identity and function. Nat Rev Immunol. 2015;15(1):7‐17. 25534619 10.1038/nri3777

[22] Youn JI , Kumar V , Collazo M , et al. Epigenetic silencing of retinoblastoma gene regulates pathologic differentiation of myeloid cells in cancer. Nat Immunol. 2013;14(3):211‐220. 23354483 10.1038/ni.2526 PMC3578019

[23] Li B , Carey M , Workman JL . The role of chromatin during transcription. Cell. 2007;128(4):707‐719. 17320508 10.1016/j.cell.2007.01.015

[24] Bickmore WA , van Steensel B . Genome architecture: domain organization of interphase chromosomes. Cell. 2013;152(6):1270‐1284. 23498936 10.1016/j.cell.2013.02.001

[25] Reddy KL , Zullo JM , Bertolino E , Singh H . Transcriptional repression mediated by repositioning of genes to the nuclear lamina. Nature. 2008;452(7184):243‐247. 18272965 10.1038/nature06727

[26] van Steensel B , Belmont AS . Lamina‐associated domains: links with chromosome architecture, heterochromatin, and gene repression. Cell. 2017;169(5):780‐791. 28525751 10.1016/j.cell.2017.04.022 PMC5532494

[27] Naetar N , Korbei B , Kozlov S , et al. Loss of nucleoplasmic LAP2alpha‐lamin A complexes causes erythroid and epidermal progenitor hyperproliferation. Nat Cell Biol. 2008;10(11):1341‐1348. 18849980 10.1038/ncb1793

[28] Shin JW , Spinler KR , Swift J , Chasis JA , Mohandas N , Discher DE . Lamins regulate cell trafficking and lineage maturation of adult human hematopoietic cells. Proc Natl Acad Sci USA. 2013;110(47):18892‐18897. 24191023 10.1073/pnas.1304996110 PMC3839750

[29] Wang AS , Kozlov SV , Stewart CL , Horn HF . Tissue‐specific loss of A‐type lamins in the gastrointestinal epithelium can enhance polyp size. Differentiation. 2015;89(1‐2):11‐21. 25578479 10.1016/j.diff.2014.12.002

[30] Youn JI , Nagaraj S , Collazo M , Gabrilovich DI . Subsets of myeloid‐derived suppressor cells in tumor‐bearing mice. J Immunol. 2008;181(8):5791‐5802. 18832739 10.4049/jimmunol.181.8.5791 PMC2575748

[31] Movahedi K , Guilliams M , van den Bossche J , et al. Identification of discrete tumor‐induced myeloid‐derived suppressor cell subpopulations with distinct T cell‐suppressive activity. Blood. 2008;111(8):4233‐4244. 18272812 10.1182/blood-2007-07-099226

[32] Chen DS , Mellman I . Oncology meets immunology: the cancer‐immunity cycle. Immunity. 2013;39(1):1‐10. 23890059 10.1016/j.immuni.2013.07.012

[33] Zwerger M , Herrmann H , Gaines P , Olins AL , Olins DE . Granulocytic nuclear differentiation of lamin B receptor‐deficient mouse EPRO cells. Exp Hematol. 2008;36(8):977‐987. 18495328 10.1016/j.exphem.2008.03.003 PMC2547467

[34] Rowat AC , Jaalouk DE , Zwerger M , et al. Nuclear envelope composition determines the ability of neutrophil‐type cells to passage through micron‐scale constrictions. J Biol Chem. 2013;288(12):8610‐8618. 23355469 10.1074/jbc.M112.441535 PMC3605679

[35] Lara‐Astiaso D , Weiner A , Lorenzo‐Vivas E , et al. Immunogenetics. Chromatin state dynamics during blood formation. Science. 2014;345(6199):943‐949. 25103404 10.1126/science.1256271 PMC4412442

[36] Yamanaka R , Barlow C , Lekstrom‐Himes J , et al. Impaired granulopoiesis, myelodysplasia, and early lethality in CCAAT/enhancer‐binding protein epsilon‐deficient mice. Proc Natl Acad Sci USA. 1997;94(24):13187‐13192. 9371821 10.1073/pnas.94.24.13187 PMC24284

[37] Karsunky H , Zeng H , Schmidt T , et al. Inflammatory reactions and severe neutropenia in mice lacking the transcriptional repressor Gfi1. Nat Genet. 2002;30(3):295‐300. 11810106 10.1038/ng831

[38] Condamine T , Mastio J , Gabrilovich DI . Transcriptional regulation of myeloid‐derived suppressor cells. J Leukoc Biol. 2015;98(6):913‐922. 26337512 10.1189/jlb.4RI0515-204R PMC4661041

