# Supplementary figures and images for: Loss of myeloid‐specific lamin A/C drives lung metastasis through Gfi‐1 and C/EBPε‐mediated granulocytic differentiation
Source: Mol Carcinog. 2020 Jan 7;59(7):679–90. doi: 10.1002/mc.23147 (PMC7282947; doi:10.1002/mc.23147)

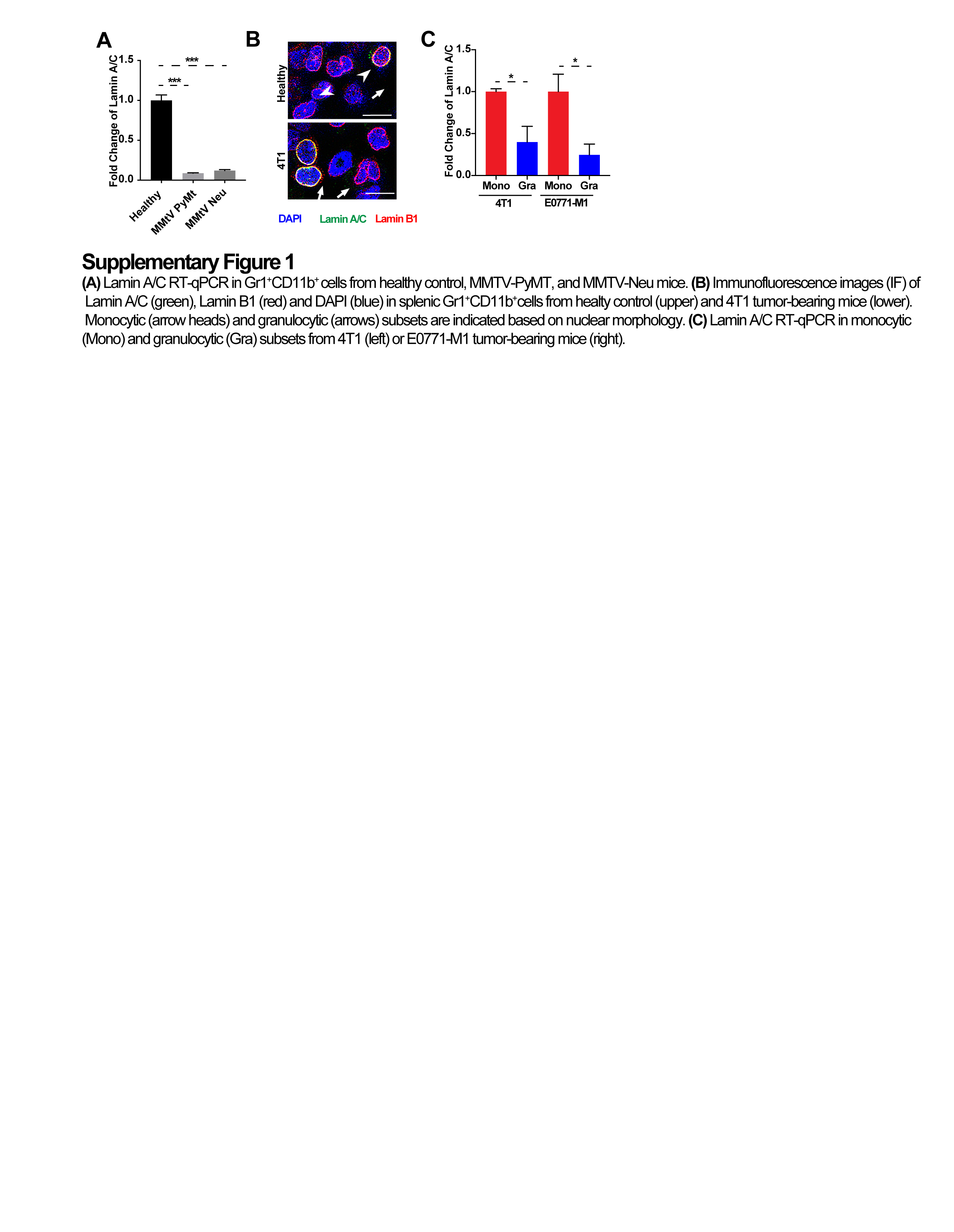

Supplement: Supplementary file 1 — Supporting information [file MC-59-679-s001.tif]

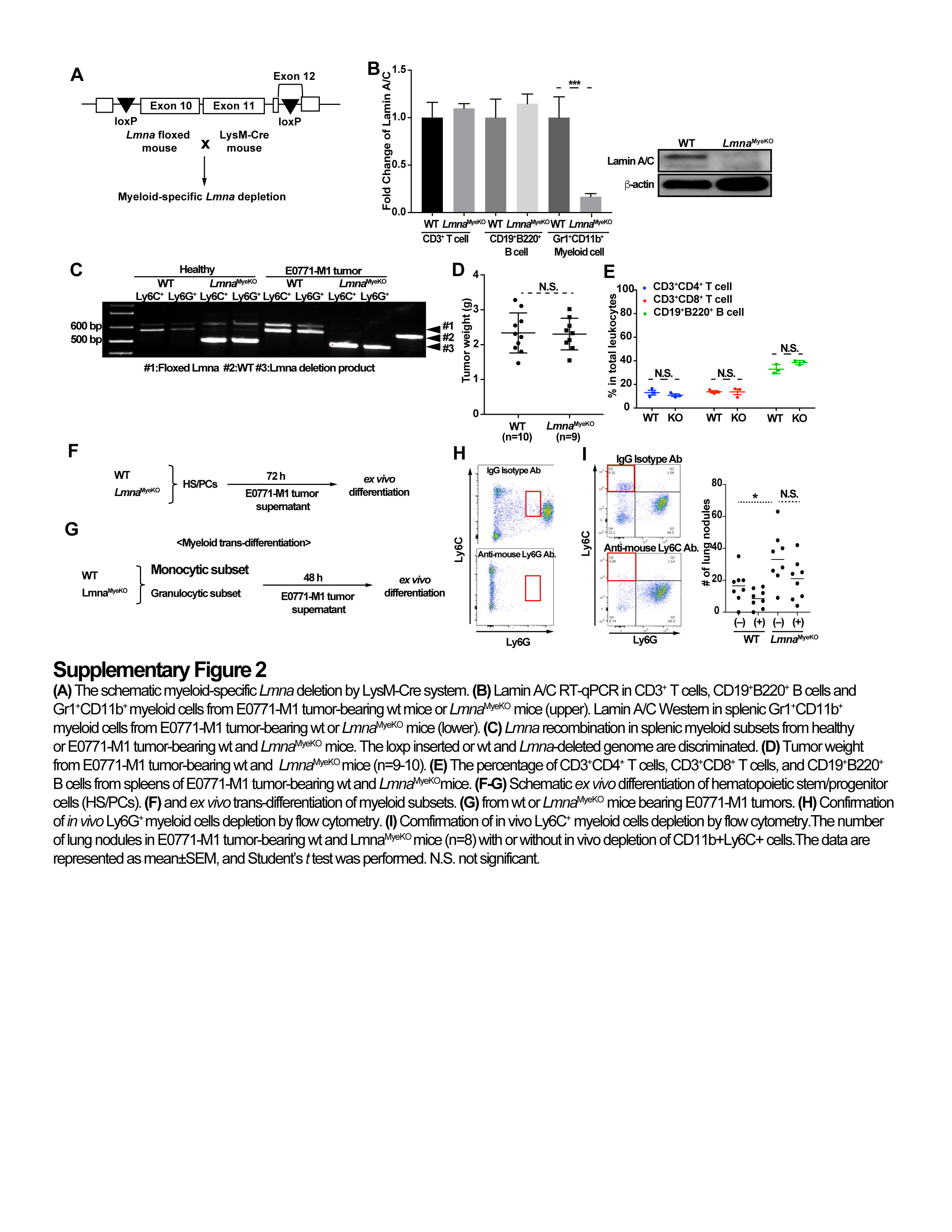

Supplement: Supplementary file 2 — Supporting information [file MC-59-679-s002.tif]

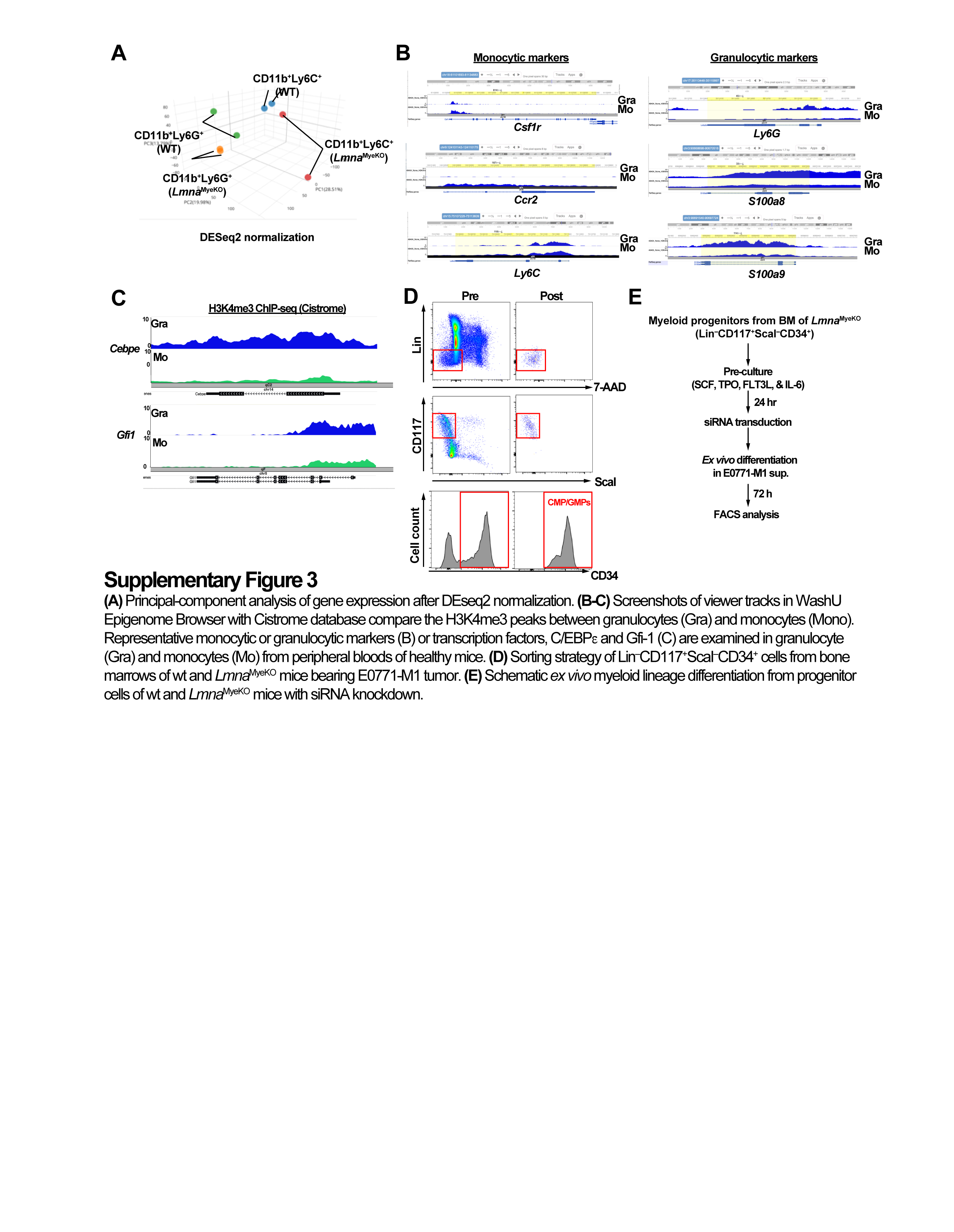

Supplement: Supplementary file 3 — Supporting information [file MC-59-679-s003.tif]

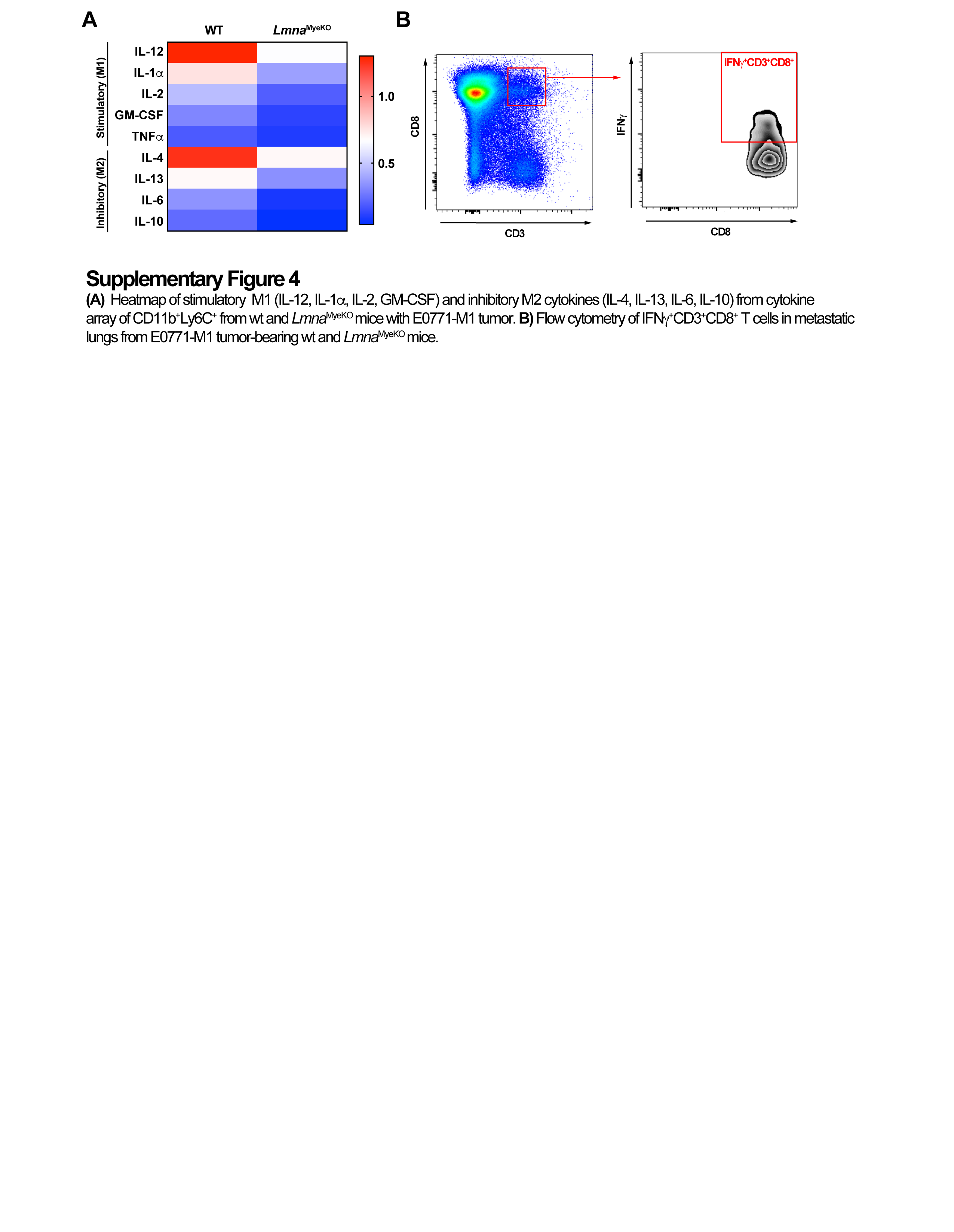

Supplement: Supplementary file 4 — Supporting information [file MC-59-679-s004.tif]

**Supplementary Table 3:**

**Sequence information**


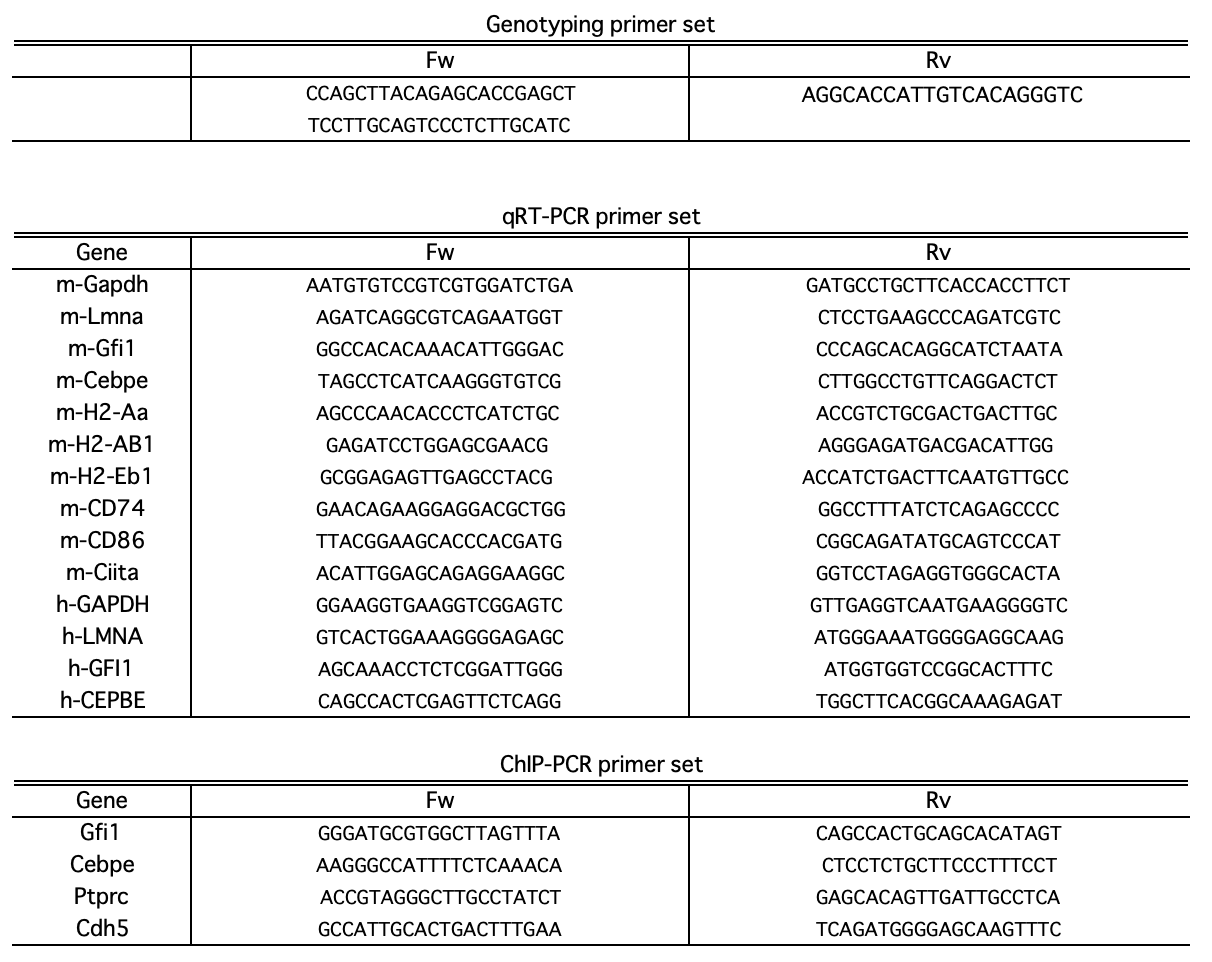

Supplement: Supplementary file 7 — Supporting information [file MC-59-679-s007.docx]
