# Supplementary material for: Loss of myeloid‐specific lamin A/C drives lung metastasis through Gfi‐1 and C/EBPε‐mediated granulocytic differentiation
Source: Mol Carcinog. 2020 Jan 7;59(7):679–90. doi: 10.1002/mc.23147 (PMC7282947; doi:10.1002/mc.23147)
Supplement: Supplementary file 5 — Supporting information [file MC-59-679-s005.docx]

**Supplementary Table 1**:

Increased genes in Lamin A/C-deficient monocytic myeloid cells compared with wt-monocytic myeloid cells.

| **Gene** | **p-value** | **Fold Change (Lmna KO/WT)** |
| --- | --- | --- |
| 1700010I14Rik | 0.037269163 | 1.637581103 |
| Abcc2 | 0.023805134 | 3.819054468 |
| Abcc4 | 0.040462274 | 1.342327276 |
| Acap3 | 0.020942603 | 1.404786267 |
| Acly | 0.030404822 | 1.290745552 |
| Adora2b | 0.049279253 | 3.173595605 |
| Ahnak | 0.005496783 | 2.162361193 |
| AI839979 | 0.017929809 | 1.548630994 |
| Aifm2 | 0.039740692 | 1.474714824 |
| Alpk1 | 0.003562429 | 1.814151392 |
| Amotl2 | 0.006627229 | 1.63530439 |
| Ampd2 | 0.007341296 | 1.409067521 |
| Anln | 0.015576768 | 1.952429245 |
| Apba1 | 0.04230527 | 2.147383011 |
| Aprt | 0.006021101 | 1.475541711 |
| Arsb | 0.011704311 | 1.658904521 |
| Asf1b | 0.035847095 | 1.998399971 |
| Atp10a | 0.025587984 | 1.91335867 |
| Atp2a3 | 0.035884281 | 1.269277079 |
| Baiap3 | 0.035366989 | 1.65701566 |
| C3 | 0.028272919 | 1.39827478 |
| Camkk2 | 0.003692544 | 1.550930427 |
| Capn2 | 0.037429265 | 1.851535896 |
| Ccdc109b | 0.034464617 | 2.268275481 |
| Ccl9 | 0.009467812 | 2.170155313 |
| Ccnb2 | 0.028289589 | 2.073349952 |
| Ccnf | 0.042299984 | 1.991921675 |
| Cd300lg | 0.02694446 | 2.664804175 |
| Cdc25a | 0.006542862 | 1.949935322 |
| Cdc45 | 0.010641299 | 1.699544423 |
| Cdca8 | 0.041554608 | 1.981796525 |
| Cdk2 | 0.021416345 | 1.330802819 |
| Cdkn2c | 0.040147742 | 1.889539333 |
| Cebpe | 0.016457189 | 1.495512444 |
| Cenpj | 0.01387855 | 1.43612144 |
| Ces1d | 2.23E-07 | 9.793574286 |
| Chaf1a | 0.046987377 | 1.959001934 |
| Chdh | 0.017254884 | 1.501744419 |
| Chil3 | 0.039390492 | 1.395015855 |
| Clspn | 0.049977504 | 2.038199508 |
| Ctbp2 | 0.039969004 | 1.451666489 |
| Ctdspl | 0.012276416 | 1.864161721 |
| Cxadr | 0.03774243 | 1.904477817 |
| Dapk1 | 0.034616797 | 1.541699944 |
| Dhcr24 | 0.006246073 | 2.127569002 |
| Dna2 | 0.01048415 | 1.761371476 |
| Dnah8 | 0.040443304 | 1.50572804 |
| E2f7 | 0.022926246 | 2.495133077 |
| E2f8 | 0.023889899 | 1.708078855 |
| Emilin1 | 0.023987748 | 1.800827844 |
| Fam129b | 0.006240313 | 2.667136931 |
| Far2 | 0.042992818 | 1.728081679 |
| Fcnb | 0.031395049 | 3.36078452 |
| Fdps | 0.003228628 | 1.865022503 |
| Ffar2 | 0.015986852 | 1.489590377 |
| Flt1 | 0.031567812 | 2.143899222 |
| Foxm1 | 0.022328918 | 2.019974807 |
| Foxred2 | 0.000620583 | 1.693742016 |
| Galns | 0.015457764 | 1.413572048 |
| Gas7 | 0.001599124 | 1.524351473 |
| Gfi1 | 0.030344403 | 1.681886786 |
| Glt1d1 | 0.021835992 | 3.559844875 |
| Gm10182 | 0.035596644 | 1.428983192 |
| Gm10282 | 0.035291137 | 1.448358397 |
| Gpc1 | 0.00733003 | 1.534072752 |
| Gys1 | 0.016107731 | 1.419150677 |
| Hmgn2 | 0.0236121 | 1.415813556 |
| Hopx | 0.042164895 | 1.345004109 |
| Hpse | 0.004685236 | 2.419726514 |
| Ica1 | 0.000570078 | 1.844713606 |
| Ifi205 | 0.024977988 | 2.332972282 |
| Ifi211 | 0.01725318 | 2.070162945 |
| Igkv4-61 | 8.83E-07 | 4.14838417 |
| Impa2 | 0.039453367 | 1.366091614 |
| Incenp | 0.027560567 | 1.57672691 |
| Inpp5j | 0.022947284 | 1.633822025 |
| Iqgap2 | 0.000966502 | 1.591654618 |
| Iqgap3 | 0.034299231 | 2.088036305 |
| Itga1 | 0.017702771 | 2.703014198 |
| Kcnab2 | 0.01062118 | 1.387575321 |
| Kif22 | 0.048487205 | 1.898933239 |
| Kif2c | 0.026848334 | 2.108918005 |
| Kifc1 | 0.031789669 | 1.97509706 |
| Kifc3 | 0.042457593 | 2.011942683 |
| Kit | 0.022652338 | 1.55034195 |
| Klrb1c | 0.045080693 | 2.012386574 |
| Kmt5c | 0.014029413 | 1.39366671 |
| Kntc1 | 0.048035054 | 1.953347169 |
| Lbp | 0.012925793 | 1.446498398 |
| Ldhb | 0.00821741 | 2.812307429 |
| Ldlrad3 | 0.006023449 | 2.486446821 |
| Lmnb2 | 0.013290279 | 1.902370329 |
| Lmo1 | 0.003931494 | 1.923652945 |
| Lonrf3 | 0.02777095 | 2.147145369 |
| Lta4h | 0.00224503 | 1.498243565 |
| Ly75 | 0.043587338 | 1.356951975 |
| Lyl1 | 0.005633257 | 1.428871397 |
| Mapkapk3 | 0.044485333 | 1.30948371 |
| Mbnl3 | 0.029590106 | 1.402802375 |
| Mfsd13a | 0.01414694 | 1.712761214 |
| Mfsd7a | 0.015026686 | 2.644873943 |
| Mgl2 | 0.005736868 | 4.756673153 |
| Mlec | 0.02101272 | 1.30797864 |
| Mlst8 | 0.039689955 | 1.373644853 |
| Mogat2 | 0.001200269 | 2.421661301 |
| Msantd3 | 0.009320985 | 2.075773257 |
| Msr1 | 0.006347961 | 2.362146517 |
| Naaa | 0.01789941 | 1.674822428 |
| Naip6 | 0.006131328 | 1.558666607 |
| Nat8l | 0.029173382 | 1.468057484 |
| Ncapd3 | 0.040316099 | 1.620735436 |
| Nedd4 | 0.005595804 | 1.364931701 |
| Neto2 | 0.015315459 | 2.537779795 |
| Nqo2 | 0.027573396 | 1.388802921 |
| Nrg1 | 0.015377736 | 2.53505009 |
| Olr1 | 0.006239683 | 1.597659932 |
| P2rx1 | 0.003957816 | 1.802660356 |
| Pafah2 | 0.043896945 | 1.461658156 |
| Papss2 | 0.049838396 | 2.112536205 |
| Phka2 | 0.017008231 | 1.471096994 |
| Pif1 | 0.023350145 | 2.131044328 |
| Plac8 | 0.030439826 | 1.740488981 |
| Plcb1 | 0.012024612 | 3.374905753 |
| Plekhg1 | 0.033368785 | 1.459073149 |
| Plk4 | 0.048688334 | 1.901394431 |
| Plscr3 | 0.029500969 | 1.548740447 |
| Plxnd1 | 0.000645065 | 2.34097464 |
| Prg4 | 0.009468956 | 4.213162649 |
| Prodh | 0.018496755 | 2.269080654 |
| Prom1 | 0.027622682 | 1.42118083 |
| Prss57 | 0.010555674 | 2.063899259 |
| Ptgr1 | 0.024037011 | 1.976648553 |
| Ptpro | 0.002686903 | 2.828871236 |
| Pxylp1 | 0.003279158 | 1.65921079 |
| Rad54l | 0.03962498 | 2.120587815 |
| Reep4 | 0.046499854 | 1.402655372 |
| Rfx2 | 0.022539162 | 1.770567198 |
| Rgcc | 0.044962868 | 1.60144642 |
| Rnpep | 0.037860161 | 1.341271713 |
| Rtkn2 | 0.030314891 | 1.718998708 |
| S100a10 | 0.043284575 | 1.816875926 |
| Serpinb10 | 0.01539855 | 1.591039421 |
| Sestd1 | 0.01928977 | 2.601969156 |
| Sh3bp1 | 0.014089868 | 1.339852495 |
| Sh3tc1 | 0.049792663 | 1.41619368 |
| Shcbp1 | 0.040869741 | 2.295033015 |
| Sipa1l3 | 0.036538342 | 1.358527468 |
| Ska3 | 0.040297768 | 2.320714358 |
| Slc35c1 | 0.011681333 | 1.431891983 |
| Slc39a11 | 0.004680353 | 1.502784412 |
| Slfn9 | 0.009394205 | 2.165143991 |
| Smtn | 0.004930964 | 1.960079674 |
| Spag5 | 0.030089478 | 2.100388583 |
| St3gal2 | 0.016102745 | 1.435802308 |
| Stil | 0.038940427 | 2.124584629 |
| Stk16 | 0.042295371 | 1.308324989 |
| Stom | 0.007215878 | 1.972395291 |
| Stx3 | 0.013990155 | 2.262024153 |
| Stxbp6 | 0.00206403 | 4.593856501 |
| Sulf2 | 0.002526376 | 2.21265867 |
| Sun1 | 0.04361451 | 1.349875137 |
| Syne3 | 0.045801163 | 1.385608493 |
| Tacc2 | 0.031265216 | 1.91736803 |
| Tbc1d24 | 0.001139569 | 1.727494822 |
| Tcf19 | 0.041245983 | 1.864786294 |
| Tesc | 0.026497406 | 1.679457491 |
| Tiam1 | 0.000112091 | 1.910171502 |
| Tmem216 | 0.031159876 | 1.419705675 |
| Tom1 | 0.022303672 | 1.389380773 |
| Tpx2 | 0.037192952 | 1.941380784 |
| Trio | 0.020505363 | 1.723734877 |
| Tshr | 0.046044541 | 1.961605956 |
| Tspan5 | 0.046763581 | 1.374077013 |
| Ttc21a | 0.037390454 | 2.762140816 |
| Tuba4a | 0.025078148 | 1.361079546 |
| Tuba8 | 0.013463105 | 1.544180985 |
| Vim | 0.016692332 | 1.344006897 |
| Xist | 2.87E-06 | 1487.738164 |
| Zfp414 | 0.019933512 | 1.417911575 |
| Zfyve9 | 0.004594515 | 2.707846259 |
