# Supplementary material for: Loss of myeloid‐specific lamin A/C drives lung metastasis through Gfi‐1 and C/EBPε‐mediated granulocytic differentiation
Source: Mol Carcinog. 2020 Jan 7;59(7):679–90. doi: 10.1002/mc.23147 (PMC7282947; doi:10.1002/mc.23147)
Supplement: Supplementary file 6 — Supporting information [file MC-59-679-s006.docx]

**Supplementary Table 1**:

Increased genes after intersected with H3K4me3 ChIP-seq analysis.

| **Gene** | **chr** | **Start** | **End** | **Fold (Granulo/Mono)** | **p-value** |
| --- | --- | --- | --- | --- | --- |
| 1700010I14Rik | chr17 | 8988225 | 8988725 | 2.5668518 | 0.000157 |
| Acap3 | chr4 | 155892341 | 155892841 | 1.65863909 | 0.012 |
| Arsb | chr13 | 93772085 | 93772585 | 1.79005014 | 0.00488 |
| Camkk2 | chr5 | 122769168 | 122769668 | 6.06286627 | 0.00138 |
| Cebpe | chr14 | 54711515 | 54712015 | 17.6304819 | 0.00324 |
| Chaf1a | chr17 | 56040182 | 56040682 | 1.85317612 | 0.00504 |
| Chil3 | chr3 | 106166601 | 106167101 | 4.46914855 | 0.000133 |
| Cxadr | chr16 | 78301805 | 78302305 | 7.2100037 | 5.83E-10 |
| E2f8 | chr7 | 48881233 | 48881733 | 2.12874036 | 6.64E-05 |
| Gfi1 | chr5 | 107724951 | 107725451 | 3.09512999 | 7.35E-05 |
| Hmgn2 | chr4 | 133966958 | 133967458 | 1.75321144 | 0.00464 |
| Ica1 | chr6 | 8770927 | 8771427 | 22.3158987 | 0.000767 |
| Impa2 | chr18 | 67299731 | 67300231 | 9.71355908 | 0.00142 |
| Inpp5j | chr11 | 3504342 | 3504842 | 5.77571678 | 0.000603 |
| Kit | chr5 | 75574964 | 75575464 | 4.34693945 | 7.90E-08 |
| Lta4h | chr10 | 93453229 | 93453729 | 1.72907446 | 9.00E-04 |
| Ly75 | chr2 | 60382841 | 60383341 | 3.03143313 | 4.82E-06 |
| Mapkapk3 | chr9 | 107279985 | 107280485 | 3.94493082 | 9.42E-06 |
| Mbnl3 | chrX | 51204644 | 51205144 | 1.905276 | 0.0128 |
| Mogat2 | chr7 | 99238019 | 99238519 | 3.63007662 | 0.00725 |
| Nedd4 | chr9 | 72662276 | 72662776 | 4.62675274 | 1.18E-08 |
| Olr1 | chr6 | 129506906 | 129507406 | 30.064728 | 2.40E-06 |
| Plekhg1 | chr10 | 3740801 | 3741301 | 1.98618499 | 0.00306 |
| Prom1 | chr5 | 44100516 | 44101016 | 24.2514651 | 3.93E-12 |
| Tesc | chr5 | 118028009 | 118028509 | 15.242208 | 6.70E-09 |
| Tmem216 | chr19 | 10555686 | 10556186 | 1.905276 | 0.00143 |
| Tshr | chr12 | 91400470 | 91400970 | 14.0256915 | 4.58E-14 |
| Tuba4a | chr1 | 75218411 | 75218911 | 1.93187266 | 6.86E-05 |
